# Progression from ductal carcinoma in situ to invasive breast cancer: molecular features and clinical significance

**DOI:** 10.1038/s41392-024-01779-3

**Published:** 2024-04-03

**Authors:** Jing Wang, Baizhou Li, Meng Luo, Jia Huang, Kun Zhang, Shu Zheng, Suzhan Zhang, Jiaojiao Zhou

**Affiliations:** 1 The Key Laboratory of Cancer Prevention and Intervention, China National Ministry of Education, the Second Affiliated Hospital, Zhejiang University School of Medicine, Hangzhou, China; 2 Department of Breast Surgery and Oncology, the Second Affiliated Hospital, Zhejiang University School of Medicine, Hangzhou, China; 3 Zhejiang Provincial Clinical Research Center for Cancer, Hangzhou, China; 4 Department of Pathology, the Fourth Affiliated Hospital, Zhejiang University School of Medicine, Yiwu, China; 5 Department of Plastic Surgery, the Second Affiliated Hospital, Zhejiang University School of Medicine, Hangzhou, China; 6 Cancer Center, Zhejiang University, Hangzhou, China

**Keywords:** Breast cancer, Breast cancer

## Abstract

Ductal carcinoma in situ (DCIS) represents pre-invasive breast carcinoma. In untreated cases, 25–60% DCIS progress to invasive ductal carcinoma (IDC). The challenge lies in distinguishing between non-progressive and progressive DCIS, often resulting in over- or under-treatment in many cases. With increasing screen-detected DCIS in these years, the nature of DCIS has aroused worldwide attention. A deeper understanding of the biological nature of DCIS and the molecular journey of the DCIS-IDC transition is crucial for more effective clinical management. Here, we reviewed the key signaling pathways in breast cancer that may contribute to DCIS initiation and progression. We also explored the molecular features of DCIS and IDC, shedding light on the progression of DCIS through both inherent changes within tumor cells and alterations in the tumor microenvironment. In addition, valuable research tools utilized in studying DCIS including preclinical models and newer advanced technologies such as single-cell sequencing, spatial transcriptomics and artificial intelligence, have been systematically summarized. Further, we thoroughly discussed the clinical advancements in DCIS and IDC, including prognostic biomarkers and clinical managements, with the aim of facilitating more personalized treatment strategies in the future. Research on DCIS has already yielded significant insights into breast carcinogenesis and will continue to pave the way for practical clinical applications.

## Introduction

Ductal carcinoma in situ (DCIS) is a stage 0 breast cancer characterized by the abnormal proliferation of epithelial cells within the ductal-lobular system of the breast. It accounts for approximately 20% cases of newly diagnosed breast cancer.^1–3^ Based on architectural patterns, DCIS has been classified into comedo, papillary, solid, cribriform, and micropapillary subtypes.^4^ Based on the histopathologic nuclear features, DCIS can present as a spectrum, with low-grade (I), intermediate-grade (II), and high-grade (III) lesions; the latter are associated with a higher likelihood of invasive ductal carcinoma (IDC).^5,6^ DCIS is commonly considered as a direct precursor to IDC. Studies have reported that approximately 25–60% of untreated DCIS cases progressed to IDC within 9–24 years of follow-up, based on the limited sample size statistics.^7–10^ However, the natural history and definite etiology of these two disease classifications remain poorly understood. Nevertheless, the development of novel technologies has offered new insights into these lesions. In this context, discoveries related to the initiation and progression of DCIS and IDC are essential for further investigation into their origin and clinical management.

DCIS is often categorized as non-invasive or pre-invasive stage of breast cancer. Nonetheless, our understanding of the underlying causes of DCIS as well as how it progressed to be invasive is limited.^11,12^ Notably, similarities and differences have been observed between DCIS and IDC. Over the years, the rapid increase in the incidence of DCIS has accompanied the widespread adoption of mammography (mostly based on screening-detected calcifications).^13–15^ In this context, mammographic calcification is more frequently detected in DCIS than in IDC. Once IDC presents with calcifications on mammography, it is more likely to be associated with synchronous high-grade DCIS.^16^ The histologic characteristic that primarily distinguishes between DCIS and IDC is that DCIS tumor cells remain confined to the mammary ductal-lobular system without invading the surrounding parenchyma and the myoepithelial layer and basement membrane are intact, while IDC tumor cells have escaped the myoepithelial layer and spread into surrounding tissues.^17^ Based on the molecular features, some studies have categorized DCIS into four intrinsic subtypes similar to those of IDC; these include luminal A, luminal B, human epidermal growth factor receptor 2 (HER2/ERBB2)-positive, and basal-like subtypes. However, there is a variation in prevalence, as the HER2-positive subtype is more commonly observed in DCIS than in IDC (approximately 35% versus 15–20%, respectively).^18–20^ Notably, different intrinsic subtypes of DCIS have been reported to be associated with distinct tumor microenvironments (TMEs) and evolutionary pathways compared to IDC.^21^ Studies have shown that DCIS and IDC share certain risk factors that contribute to their incidence; these include age, family history, breast density, and hormone therapy.^22^ However, it is commonly considered that the prognosis of DCIS is superior to IDC. DCIS is not considered a life-threatening disease and is linked to a high rate of overall survival and a normal life expectancy.^23^ After treatments, the overall recurrence rate of DCIS is approximately 20%, in which 50% are in situ recurrence while another 50% invasive recurrence.^24^ It is therefore essential to identify the initiation of these lesions and the relationships between them; in particular, the molecular events underlying progression from DCIS to IDC warrant further investigation.

**Fig. 1:**
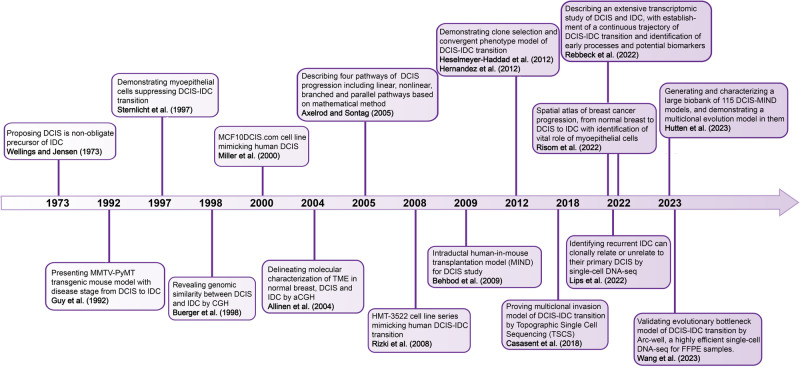
Highlights of DCIS-IDC research. Since DCIS was termed as the precursor of IDC in 1973, research on the transition from DCIS to IDC has rapidly advanced. This progress has been fueled by the establishment of in vitro DCIS cell lines and in vivo animal models. In recent years, technological advancements have offered deeper insights into the changes occurring in both tumor cells and the microenvironment during the transition from DCIS to IDC

Although the epidemiological and clinicopathological characteristics indicate the progression from DCIS to IDC, the underlying biological mechanisms remain obscure. Here, we review the highlights of DCIS-IDC studies (Fig. 1). Since DCIS was termed as the precursor of IDC in 1973,^25^ studies on DCIS-IDC transition have been rapidly evolving since the establishment of DCIS cell lines and in vivo models. Recent advancements in technology, such as single-cell sequencing, spatial transcriptomics, and artificial intelligence, have provided deeper insights into the molecular changes occurring during the transition from DCIS to IDC. These studies have illuminated the alterations in both tumor-intrinsic features and the surrounding microenvironment. However, different studies relied on a variety of the sample populations and diverse techniques, making it necessary to systematically review all these related studies to get a clear picture of the known precise biological mechanisms underlying DCIS-IDC transition.^26^ Moreover, the clinical and pathological markers that are currently used to predict prognosis mostly rely on a combination of factors including patient age, surgical margins, tumor size, and nuclear grade, however, all these factors fail to predict prognosis independently with high confidence.^27–30^ Thus, the question whether current treatment is overly aggressive for indolent DCIS or insufficient for progressive DCIS remains unanswered. This ongoing debate highlights the need for further research and a better understanding of the natural history and biology of DCIS in order to develop personalized and targeted management approaches for each individual patient.

This review highlights the pivotal signaling pathways in breast cancer and subsequently delves into the molecular distinctions between DCIS and IDC. It offers valuable insights into the biology of the transition from DCIS to IDC, shedding light on notable alterations occurring in tumor cells and the surrounding microenvironment. Essential experimental models and advanced technologies that are used for studies on DCIS-IDC have also been discussed. In addition, the recent clinical advances in DCIS and IDC, including prognostic biomarkers and advanced treatments have been described. Finally, this review aimed to identify the biology of the origin and progression of DCIS in order to better individualize treatments for DCIS with variable malignant potential.

## Key signaling pathways in breast cancer

**Fig. 2:**
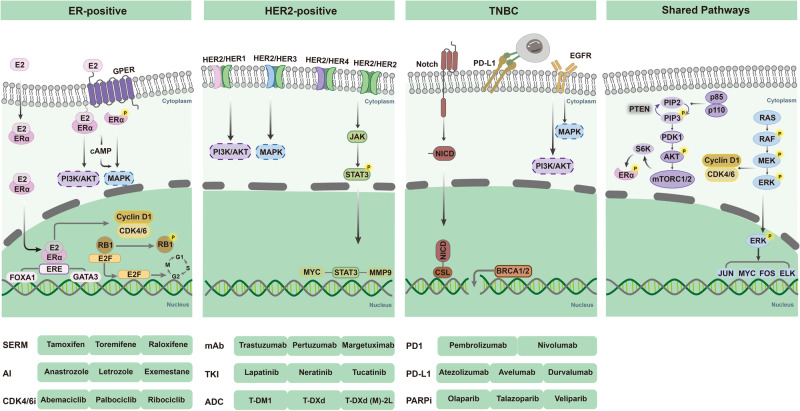
Key signaling pathways in breast cancer. Breast cancer is a heterogeneous disease characterized by diverse subtypes. The development and progression of breast cancer result from the influence of subtype-specific and shared signaling pathways, as well as the intricate crosstalk between them. Inhibitors that target these key signaling pathways have led to improvement in the prognosis of breast cancer. E2 estradiol, ER estrogen receptor, ERE estrogen response element, GPER G protein-coupled ER, HER2 human epidermal growth factor receptor 2, TNBC triple-negative breast cancer, EGFR epidermal growth factor receptor, NICD Notch intracellular domain, SERM selective ER modulator, AI aromatase inhibitor, CDK4/6i cyclin-dependent kinase 4/6 inhibitor, mAb monoclonal antibody, TKI tyrosine kinase inhibitor, ADC antibody–drug conjugates, PARPi Poly (ADP‑ribose) polymerase inhibitor

Why DCIS attracts much attention although itself is not invasive? It is probably because that DCIS can progress to IDC. IDC represents 60–75% of invasive breast cancer cases and breast cancer remains a leading cause of cancer-related mortality in women.^31^ Breast cancer is heterogeneous. IDC of different subtypes shared some common oncogenic pathways, as well as being perturbed by dominant pathways distinctively. The most important pathways acknowledged in breast cancer including estrogen receptor (ER) pathway,^32,33^ HER2 signaling pathway,^34,35^ Phosphoinositide 3-kinase/protein kinase B/mammalian target of rapamycin (PI3K/AKT/mTOR) pathway,^36^ mitogen-activated protein kinases (MAPK) pathway,^36,37^ and cyclin D1/cyclin-dependent kinase 4/6/retinoblastoma protein (cyclin D1/CDK4/6/RB1) pathway (Fig. 2). Metastasis retains a significant problem of breast cancer, with 20–30% of patients in early-stage breast cancer still die of metastatic disease.^38^ The metastatic cascade is admitted as a multistep process^39^ and the route of disseminated breast cancer cell to metastatic success or failure is decided by tumor cell intrinsic factors such as genetic/epigenetic plasticity, stemness, epithelial–mesenchymal transition and the tumor cell extrinsic factors especially including the metastatic microenvironment and the anti-cancer drug action.^40–42^

### Estrogen receptor pathway

Hormone exposure is served as the primary risk factor for sporadic breast cancer.^43^ The two main steroid hormones involved in breast cancer are estrogen and progesterone, which are linked to the growth and proliferation of breast cells. Higher hormone exposure increases the risk of breast cancer, including shorter menstrual cycle, early menarche, and late menopause.^44^ Estrogen is regarded as a major promoter of breast cancer, especially for ER-positive breast cancer subtype, and the most common type in human is 17β-estradiol (E2).^45^ The binding of E2 to estrogen receptors (ERs, mostly ERα, encoded by the *ESR1* gene) stimulates the classical genomic ER signaling pathway, which ultimately contributes to the increase of breast cancer cells’ proliferation and decrease of their apoptosis.^46^ More specifically, when activated by E2 binding, the estrogen-ER dimer translocates to the cell nucleus and engages with coregulator proteins and specific DNA sequences known as estrogen responsive elements (EREs). These interactions further modulate the expression of downstream genes such as GATA3 and FOXA1 that participate in breast carcer initiation, progression, and metastasis (Fig. 2).^47,48^ On the other hand, a nongenomic ER signaling pathway involves a membrane-anchored G protein-coupled estrogen receptor (GPER). Activation of GPER triggers signaling cascades such as PI3K/AKT and Ras/MAPK, which in turn regulate the transcription of genes involved in breast cancer development.^45,49^

### HER2 signaling pathway

HER2, encoded by the *ERBB2* gene, is one of the four members of epidermal growth factor receptor (EGFR) family, which includes EGFR (HER1), HER2, HER3, and HER4.^43,50^ HER2 is enriched in approximately 15–20% of breast cancers, which is correlated with a highly aggressive phenotype and unfavorable prognosis.^50,51^ Although HER2 has no specific ligand, it forms homodimers or heterodimers with HER1, HER3, or HER4 to initiate downstream signaling pathways (Fig. 2).^52–54^ In breast cancer, activation of HER2 signaling further triggers various downstream signalings, such as PI3K/AKT, MAPK, and JAK/STAT signaling pathways, all of which leading to cancer cell proliferation, survival, adhesion and metastasis.^50,55^ Targeting HER2 is proved to have great efficacy in the treatment of HER2-positive breast cancer patients, which profoundly benefits their overall survival.

### PI3K/AKT/mTOR pathway

The phosphatidylinositol 3-kinases (PI3Ks) are a group of intracellular kinases that are categorized into three classes (class I, II and III). Among them, class I PI3Ks composed of a regulatory (p85) and a catalytic (p110) subunit are the most commonly studied and definitely implicated in oncogenesis.^41,56^ Mutations commonly occur in the p110α subunit (encoded by *PIK3CA*), among all breast cancer subtypes, but the mutation frequency is especially higher in ER-positive breast cancers. Approximately, mutations in *PIK3CA* are observed in 40% of ER-positive breast cancers, 25% of HER2-positive breast cancers, and 9% of TNBC.^56^ *PIK3CA* mutations lead to the activation of PI3K, which activates downstream targets such as protein kinase B (AKT) and mammalian target of rapamycin (mTOR). The excessive activation of the PI3K/AKT/mTOR pathway is strongly linked to uncontrolled breast cancer development.^57^ Phosphatase and tensin homolog (PTEN) and inositol polyphosphate 4-phosphatase type II (INPP4B) are two crucial negative regulators of the PI3K pathway (Fig. 2). In breast cancer, there is often a decrease in the expression of PTEN and INPP4B, further enhancing the activation of the PI3K/AKT/mTOR pathway,^58–60^ and dysregulation of this signaling pathway is recognized as a mechanism of resistance to endocrine and anti-HER2-targeted treatment.^61–63^ Consequently, targeting PI3K/AKT/mTOR pathway has emerged as a promising approach for precise therapeutic intervention in breast cancer.

### MAPK pathway

Mitogen-activated protein kinases (MAPKs) are phosphoproteins stimulated by mitogens and play a vital role in controlling various cellular processes such as cell proliferation, stress adaptation, differentiation, and apoptosis. Mammalian cells have three major branches of the MAPK signaling pathways: the extracellular signal-regulated kinases (ERK), the c-Jun N-terminal kinases (JNK), and the p38 MAPKs (Fig. 2). MAPKs are strongly related to breast cancer prognosis, which participate in hormone receptor modulation, response to growth factors and targeted therapies.^64–66^ The MAPK pathway is activated in approximately 50% of breast cancers.^67^ RAS mutation, which frequently observed in tumors such as pancreatic cancer and colorectal cancer,^68^ leading to constitutive activation of ERK1/2, is not considered as the main cause of MAPK signaling activation in breast cancer, as RAS mutation occurs in less than 5% of breast cancer cases. Contrarily, MAPK signaling activation in breast cancer is usually considered as a result of constitutive upstream signaling, such as the ER signaling pathway and HER2 overexpression. In ER-positive breast cancers, estrogen can stimulate growth factors such as transforming growth factor beta (TGF-β), insulin-like growth factor type 1 (IGF-1), that ultimately activate MAPK pathway.^69^ The activated MAPKs can also phosphorylate ER, either through direct or indirect signaling pathways, resulting in an sustainedly enhanced transcriptional efficiency of the receptor.^70^

### Cyclin D1/CDK4/6/RB1 pathway

In breast cancer, the cyclin D1/CDK4/6/RB1 complex plays a crucial role in cell proliferation mediated by ER signaling.^71^ Particularly, the presence of estrogen in ER-positive breast cancers induces the expression of cyclin D1(encoded by *CCDN1*), resulting in the activation of CDK4/6. CDK4/6 activity consequently leads to the hyperphosphorylation of RB1, facilitating the progression of the cell cycle and promoting cellular proliferation (Fig. 2).^72,73^ What’s more, increased MAPK and PI3K/AKT pathways can also drive CCDN1 transcription that finally activate cyclin D1/CDK4/6/RB1 pathway.^74^ CDK4/6 inhibitors are undeniably considered as one of the most significant advancements in breast cancer treatments in the last two decades.^75^ Different from ER-positive breast cancer, TNBC often exhibits a loss of RB1 expression, which consequently renders them unresponsive to CDK4/6 inhibitors.^76,77^

## Molecular features of DCIS and IDC

A traditional theory presumes that DCIS and IDC are derived from mammary ducts, while lobular carcinoma in situ and invasive lobular carcinoma arise from lobules,^78^ although in Wellings’ research, they found that most early-stage breast carcinomas including ductal and lobular types arise from the same structure, namely, the terminal duct lobular unit.^25,79^ The ductal structure of terminal duct lobular unit consists of two cell layers, namely, the epithelial cell layer within the lumen and the myoepithelial cells (MECs) layer (surrounded by basement membrane). From a morphological perspective, some DCIS can be visually distinguished from IDC on H&E slides, which is characterized by neoplastic proliferation within the terminal duct lobular unit and invariable encasement by the basement membrane,^80^ while some other small DCIS need to be differentiated from early IDC by a combination of myoepithelial markers such as cytokeratin 5/6 (CK5/6), tumor protein p63 (P63), calponin, α‐smooth muscle actin, etc.^81–83^ To date, numerous studies have explored the genomic events in DCIS and compare them with those of IDC, they have put insights into the distinctions between synchronous DCIS and IDC, pure DCIS and DCIS with synchronous IDC, and primary DCIS and recurrent DCIS or IDC (Table 1).

**Table 1:** Comparison of molecular features between DCIS and IDC

Author	Year	Samples	Analyses	Methods	Highlights
a. Synchronous DCIS VS Synchronous IDC					
Moelans et al.^90^	2011	39 synchronous DCIS-IDC	DNA	MLPA	No significant differences were found in copy numbers of 21 genes in synchronous DCIS and IDC, except BIRC5 being more prevalent in DCIS but without significance.
Hernandez et al.^84^	2012	13 synchronous DCIS-IDC	DNA	aCGH, Sequenom MassARRAY	The genomic profiles of synchronous DCIS-IDC were largely similar, but PIK3CA mutations were limited to the DCIS component in 2/13 cases, and 1/13 case exhibited a higher frequency of PIK3CA mutations in DCIS compared to IDC.
Johnson et al.^91^	2012	21 synchronous DCIS-IDC	DNA	MIP array	83% of the genome was shared in synchronous DCIS-IDC, with recurrent losses at 3q, 6q, 8p, 11q and gains at 5q, 16p, 19q and 20 observed in IDC but not in DCIS. Additionally, amplification of CCND1 and MYC was more pronounced in IDC, while loss of 17p11.2 was specific to DCIS.
Casasent et al.^92^	2018	10 synchronous DCIS-IDC	DNA	TCSC	A direct genomic lineage existed between synchronous DCIS and IDC, with most mutations and CNAs evolved within the ducts prior to invasion.
Pareja et al.^86^	2020	27 synchronous DCIS-IDC	DNA	WES, MSK-IMPACT	Genetic alterations were similar between synchronous DCIS and IDC, the most overlapped mutations include TP53, PIK3CA and GATA3.
Fortunato et al.^87^	2021	53 synchronous DCIS-IDC	DNA	DNA-seq	Statistically significant differences were identified that IDC harbored increased mutations and higher genetic divergence than synchronous DCIS.

b. Primary DCIS VS Recurrent DCIS/IDC					
Waldman et al.^95^	2000	18 primary DCIS, 18 recurrent DCIS	DNA	CGH	DCIS recurrences were clonally related to their primary DCIS in 17/18 cases. The most common chromosomal alterations shared between primary and recurrent DCIS were gains involving chromosome 17q and losses involving chromosomes 8p and 17p.
Gorringe et al.^96^	2015	8 primary DCIS, 6 recurrent DCIS, 1 IDC, 1 mixed DCIS	DNA	SNP arrays	There was no significant difference in fraction genome alterations between primarily DCIS and matched recurrent tumors, in which a large variation in the copy number altered base pairs showing the same gain or loss event ranging from 11 to 58%.
Trinh et al.^94^	2021	6 primary DCIS, 6 recurrent IDC	DNA, mRNA	WES, RNA-Seq	Amongst pure DCIS and matched recurrent IDC, frequent changes were observed including 1q, 8q, 16p, 17 amplification and loss of 11q and 16q.
Lips et al.^97^	2022	129 primary DCIS, 34 recurrent DCIS, 95 recurrent IDC	DNA	WES, Single-cell sequencing	Most of the recurrent IDC or DCIS were found clonally related to primary DCIS. The most common shared mutations between primary DCIS and recurrent IDC occur in TP53 and PIK3CA, and 1q and 8p11 gain were more common in recurrent IDC compared to primary DCIS, while 3p21 loss was more common in primary DCIS compared to recurrent IDC.
Wang et al.^98^	2023	10 primary DCIS, 10 recurrent DCIS/IDC	DNA	Arc-well	Seven samples with evolutionary bottlenecks were found, in which common CNA events of persistent subclones showed increased chr3q (PIK3CA), chr8p (MYC, CCNE2) and 20q (ZNF217, AURKA) gains in recurrent tumors comparing to primary DCIS.

c. Pure DCIS VS DCIS with synchronous IDC					
Iakovlev et al.^85^	2008	6 pure DCIS, 17 DCIS with synchronous IDC	DNA	aCGH	Pure DCIS exhibited a higher degree of genomic complexity than DCIS with synchronous IDC, in which the gain on 17q22-q24.2 was less common while the gain at 17q12-21.2 was more common in pure DCIS.
Zhou et al.^99^	2009	32 pure DCIS, 48 DCIS with synchronous IDC	DNA	DNA sequencing	TP53 mutation frequency was found slightly lower in pure DCIS (15.6%) than synchronous DCIS (20.8%).
Miron et al.^100^	2010	43 pure DCIS, 31 DCIS with synchronous IDC	DNA	Sanger sequencing	PIK3CA mutations were less common in pure DCIS (5%) than DCIS with synchronous IDC (16%).
Sakr et al.^101^	2014	89 pure DCIS, 119 DCIS with synchronous IDC	DNA	Sequenom MassARRAY	PIK3CA hotspot mutations and pAKT expression were more prevalent in ER + /HER2- DCIS with synchronous IDC, while INPP4B loss of expression was more frequent in ER-/HER2 + DCIS with synchronous IDC than pure DCIS.
Afghahi et al.^102^	2015	120 pure DCIS, 151 DCIS with synchronous IDC	DNA	FISH	Pure DCIS had lower frequencies of CNAs at three common chromosomal loci 1q, 8q24 and 11q13 than DCIS with synchronous IDC
Kim et al.^103^	2015	6 pure DCIS, 5 DCIS with synchronous IDC	DNA	aCGH, WES	Gains of PIK3CA, CDK12, MLF1, EVI1, SOX2, TFRC, ERG and MTCP1, and losses of PIK3R1, APC, FGFR2, PDGFRB, CD74, ITK, EBF1, RANBP17, TLX3, NPM1, NR4A3, IL6ST and MAP2K4 were more frequent in DCIS with synchronous than pure DCIS
Lin et al.^106^	2019	65 pure DCIS, 60 DCIS with synchronous IDC	DNA	Targeted sequencing	The mutations of PIK3CA kinase domain were found more frequent in pure DCIS.
Bergholtz et al.^104^	2020	10 pure DCIS, 13 DCIS with synchronous IDC	DNA	Targeted sequencing	A lower frequency of TP53, PIK3CA, and ERBB2 mutations was found in pure DCIS.
Rebbeck et al.^108^	2022	A whole of >2,000 ductal lesions from 145 patients	mRNA	RNA-seq	CAMK2N1, MNX1, ADCY5, HOXC11 and ANKRD22 were found reduced expression in DCIS with synchronous than pure DCIS.

*DCIS* ductal carcinoma in situ, *IDC* invasive ductal carcinoma, *CGH* comparative genomic hybridization, *MLPA* multiplex ligation-dependent probe amplification, *MIP* molecular inversion probe, *WES* whole-exome sequencing, *MSK-IMPACT* MSK Integrated Mutation Profiling of Actionable Cancer Targets, *TCSC* Topographic Single Cell Sequencing, *IHC* immunohistochemistry, *FISH* fluorescence in situ hybridization, *aCGH* array-comparative genomic hybridization, *CNAs* copy number aberrations, *Arc-well* Archival nanowell sequencing

### Comparison of synchronous DCIS and IDC

Synchronous DCIS and IDC are frequently found in patients who are diagnosed with invasive breast carcinoma. To identify the progressive markers, a number of studies have explored genomic profiles of synchronous DCIS and IDC which were usually micro-dissected in the same tumor sample. Most of these studies concluded that synchronous DCIS and IDC share high levels of genomic concordance, which is reflected by their copy number profiles or copy number aberrations (CNAs) (Table 1).^84–89^ Moelans et al. described no significant difference in copy numbers of 21 genes in synchronous DCIS and IDC.^90^ Hernandez et al. demonstrated a largely similar genomic profiles of synchronous DCIS and IDC,^84^ and Johnson et al. further depicted 83% of the genome in synchronous DCIS and IDC were shared.^91^ In a recent whole-exome sequencing study, Pareja et al. evaluated 27 formalin-fixed paraffin-embedded (FFPE) samples of synchronous DCIS and IDC, finding that the most frequent gains and losses in both synchronous DCIS and IDC were on 1q, 16p, 5q, 6q, and 8p, and the most overlapped mutations in genes included tumor protein p53 (*TP53*), phosphatidylinositol-4, 5-bisphosphate 3-kinase catalytic subunit alpha (*PIK3CA*), and GATA binding protein 3,^86^ which have been considered to play pivotal roles in breast cancer initiation and progression. In essence, given the notable genetic similarity observed between synchronous DCIS and IDC, it may be inferred that synchronous DCIS has fundamentally invasive potential, wherein invasive modifications have already occurred as early as DCIS stage. However, the limited findings of the differences between synchronous DCIS and IDC may attribute to the bottleneck of high-resolution techniques especially at single-cell level. Most recently, by using spatially-resolved single-cell DNA sequencing, Casasent et al. reported a direct genomic lineage between synchronous DCIS and IDC, with most mutations and copy number aberrations evolved within the ducts prior to invasion.^92^

However, notably, studies have also found differences between synchronous DCIS and IDC. Fortunato et al. analyzed single nucleotide variants in synchronous DCIS and IDC lesions and found significantly increased mutations and genetic divergence in IDC than synchronous DCIS.^87^ A meta analyses performed by Rane et al. found that DCIS displays more frequent losses on 5q31.1–5q35.3, 6q25.3–6q26, and 13q32.3–13q33.1 and gains on 11p12 in synchronous DCIS than synchronous IDC.^88^

In essence, given the notable genetic resemblance observed between synchronous DCIS and IDC, it can be inferred that synchronous DCIS is fundamentally invasive, wherein invasive modifications have already taken place. Although slight disparities between these two conditions have been documented, there is limited consensus regarding the genomic alterations that signify transitions. The critical events associated with the transition from DCIS to IDC may occur in earlier lesions such as pure DCIS or possibly even in lesions preceding DCIS.

### Comparison of primary DCIS and recurrent DCIS or IDC

Recurrent DCIS or IDC refers to subsequent carcinoma lesions that develop after a primary diagnosis of DCIS. Notably, up to 20% of patients with DCIS develop recurrent DCIS or IDC despite treatment and approximately half of these are IDC.^4,93^ Owing to difficulties in collecting samples from primary DCIS and their matched recurrent tumors, related data of genomics, transcriptomics, and proteomics are still limited. Recurrence is considered as the DCIS progression and comparison studies of primary pure DCIS and recurrent DCIS or IDC have been launched by the aim of exploring the biological factors and molecular features leading to DCIS progression, however limited significant alternations were founded between primary DCIS and recurrent DCIS or IDC at genetic level. Previous studies by using conventional techniques such as comparative genomic hybridization, single nucleotide polymorphism arrays or whole-exome sequencing, consistently found that primary DCIS and their recurrent tumors were genetically related.^94–96^ In recent important study by Lips et al. whole-exome and single-cell sequencing were conducted among 129 primary DCIS, 34 matched recurrent DCIS and 95 matched recurrent IDC. Most of the recurrent IDC or DCIS were found clonally related to primary DCIS. When comparing primary DCIS and recurrent IDC, the numbers of shared and private mutations were found to be highly variable for different matched tumor pairs, with most common shared mutations occurring in TP53 and PIK3CA. Besides, 1q and 8p11 gain were more common in recurrent IDC compared to primary DCIS, while 3p21 loss was more common in primary DCIS compared to recurrent IDC. When comparing primary DCIS and DCIS recurrence, whole-exome sequencing and copy number profiling data revealed 29/34 cases were related, suggesting the DCIS recurrence as the residual DCIS which was not detected by imaging preoperatively.^97^ Another recent important study by Wang et al. conducted a high-throughput single-cell DNA sequencing (Arc-well) in 10 paired archival FFPE samples of primary DCIS and recurrent tumors.^98^ Evolutionary analysis indicated that majority of DCIS cases in the cohort went through an evolutionary bottleneck. Specific chromosome aberrations were identified in the persistent subclones across the primary DCIS and recurrent tumors, which were considered to be closely related to DCIS recurrence. In the seven samples with evolutionary bottlenecks, increased chr3q (PIK3CA), chr8p (MYC, CCNE2) and 20q (ZNF217, AURKA) gains were found in the persistent subclones in recurrent tumors comparing to primary DCIS.

In summary, genomic investigations have demonstrated a close genetic connection between recurrent DCIS or IDC and their primary pure DCIS counterparts. Only minimal alternations were noted between primary DCIS and their recurrence. It indicates that genomic changes responsible for recurrence may occur as an early occurrence in primary DCIS. Based on the numerous genomic studies, landscape of transcriptomics and proteomics is required to further discover the molecular features occurring in DCIS recurrence. Besides, the influence of microenvironment in DCIS recurrence needs to be further investigated too.

### Comparison between pure DCIS and DCIS with synchronous IDC

Studies also interested in distinguishing the molecular features between pure DCIS and DCIS from patients diagnosed with co-occurring IDC, which refer to DCIS with synchronous IDC here. Many studies observed that pure DCIS exhibited lower genomic instability compared to DCIS with synchronous IDC.^99–104^ For example, in Afghahi et al.’s study with large sample size, pure DCIS was found to have lower frequencies of CNAs at three common chromosomal loci 1q, 8q24, and 11q13 than DCIS with synchronous IDC.^102^ However, there are some dissenting voices. For example, a previous study by Iakovlev et al. with limited sample size, demonstrated that pure DCIS exhibited a higher degree of genomic complexity than DCIS with synchronous IDC, in which the gain on 17q22-q24.2 was less common while the gain at 17q12-21.2 was more common in pure DCIS.^85^ Mutations of driver genes such as TP53 and PIK3CA have been identified with significant differences between pure DCIS and DCIS with synchronous IDC.^99,105–107^ For example, in Zhou et al.’s study, TP53 mutations were less common in pure DCIS than IDC with synchronous IDC.^99^ In Bergholtz et al.’s study, a lower frequency of TP53, PIK3CA, and ERBB2 mutations was found in pure DCIS comparing to DCIS with synchronous IDC. It should be noted that mutations in specific regions of driver genes should be paid more attentions to.^107^ Earlier studies by Miron et al. and Sakr et al. typically found more PIK3CA mutations in DCIS with synchronous IDC than pure DCIS.^100,103^ Contrarily, a recent study by Lin et al., by targeted exon sequencing in the kinase domain of PIK3CA, discovered that mutations in this specific domain was more frequent in pure DCIS than those in DCIS with synchronous IDC.^106^ The discrepancy may attribute to that Lin et al.‘s study focusing on a more precise mutation region in PIK3CA kinase domain, while earlier studies looked at a combination of regions in PIK3CA termed as “hotspot mutations”.

Intriguingly, Rebbeck et al. recently conducted a comprehensive transcriptomic study involving over 2,000 micro-dissected ductal lesions from 145 patients.^108^ In the study, they compared pure DCIS with concurrent DCIS which refer to DCIS with synchronous IDC and successfully identified some genes that were potentially responsible for DCIS progression, such as CAMK2N1, MNX1, ADCY5, HOXC11, and ANKRD22, which exhibited reduced expression in concurrent DCIS.

In brief, pure DCIS exhibited fewer genetic events and less frequent mutations in driver genes than DCIS with synchronous IDC, suggesting that concurrent DCIS is probably in a more progressive stage rather than pure DCIS in. Emerging studies of transcriptome in pure DCIS and DCIS with synchronous decipher distinguished related genes expression, although these studies are very limited. However, if molecular features derived from the comparisons between pure DCIS and DCIS with synchronous IDC are considered as to be responsible for DCIS progression, it should be admitted first that DCIS is the precursor of IDC.

## Progression from DCIS to IDC

Although the origin and evolution of breast cancer has been long discussed, the mechanisms involved remain unclear. In regard to tumor heterogeneity, two distinct but not mutually exclusive models were proposed, namely, the clonal evolution and cancer stem cell models.^109–112^ In the cancer stem cell model, tumor-initiating cells originate from a small fraction of stem or progenitor cells which can undergo self-renewal. In the clonal evolution model, any breast epithelial cell has tumorigenic potential and tumor progression is promoted by cumulative genetic and epigenetic changes.

However, as a key stage in breast carcinogenesis, the evolution from DCIS to IDC remains largely obscure. The major questions in this regard are: (1) does IDC originate from DCIS? and (2) if so, how does DCIS progress during the DCIS-IDC transition? The four proposed models of DCIS-IDC transition may answer the former question. The theories regarding DCIS progression have been broadly divided into two categories, namely, genetic and non-genetic. The former posits the crucial role of the neoplastic cell itself while the latter emphasizes on non-genetic factors, particularly the TME.

### Proposed models of DCIS progression

**Fig. 3:**
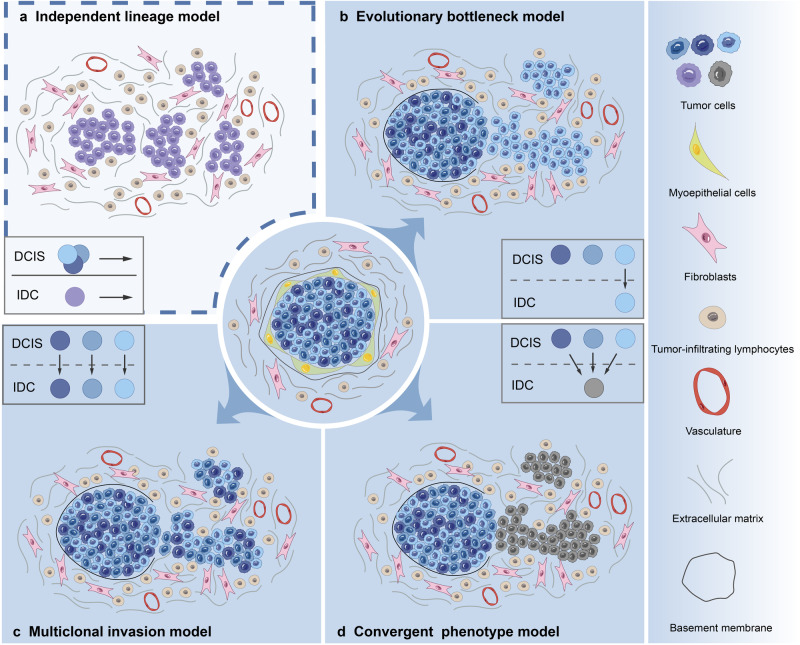
Proposed models of DCIS progression. **a** Independent lineage model, which presumes that DCIS and IDC derive from two distinct normal epithelial cells which share no overlapping CNAs or mutations. **b** Evolutionary bottleneck model, which presumes that a specific clone within DCIS is selected and it evolves into IDC. **c** Multiclonal invasion model, which presumes that multiple clones escape and co-migrate to invasive regions to generate IDC. **d** Convergent phenotype model, which presumes that subclones of different genotypes within DCIS can all give rise to an invasive phenotype to establish IDC

Four different but complementary models have been proposed for DCIS progression, including the independent lineage, evolutionary bottleneck, multiclonal invasion, and convergent phenotype models (Fig. 3).^113–115^ The independent lineage model and the other three direct lineage models differ on whether DCIS is a precursor of IDC. Naturally, only up to 60% of DCIS was observed to progress to IDC if left untreated, while at least the left 40% of DCIS remained indolent and never progressed. Thus, the existence of indolent DCIS provided evidences for independent lineage model that DCIS and IDC were of independent origin.^7^ Moreover, independent lineage model opposes the precursor theory based on the identification of discordant markers between DCIS and IDC. Contrarily, Wellings and Jensen et al. suggested that ductal breast carcinomas undergo continuous histological progression from atypical ductal hyperplasia (ADH) to DCIS and subsequently to IDC.^25^ The gradual histological continuity, similar intrinsic subtypes, and general genetic similarity between DCIS and adjacent IDC further validates the theory.^116–118^ However, low-grade and high-grade DCIS are supposed to individually progress to IDC via different pathways.^78,119^ In addition to partially explaining whether DCIS is a precursor of IDC, these models illustrate the transition from DCIS to IDC in terms of changes in the tumor cell. Extensive research using sequencing has clearly demonstrated intratumor heterogeneity in DCIS and IDC; this heralds a new era in investigations on DCIS progression.

#### Independent lineage model

The independent lineage model hypothesizes that DCIS and IDC derive from two distinct normal epithelial cells (Fig. 3a).^92^ It presumes that DCIS and IDC arise from and progress through two independent cell lineages which never share any CNAs or mutations. The model opposes the opinion that DCIS is the precursor of IDC which has been generally accepted in recent years. In support of this theory, Studies results of discordant targeted gene or protein markers and CNAs in DCIS and IDC supported the theory of independent lineage model.^91,100,120,121^ In this context, Sontag et al. used a mathematical approach and concluded that DCIS and IDC develop in parallel pathways.^122^ Johnson et al. consistently identified IDC-specific gains and losses on chromosomal regions to be restricted to IDC; this was not observed in DCIS.^91^ Moelan et al.’s study found the observed methylation of *CDKN2A* and *CHFR* to only exist in DCIS.^123^ The independent model is also supported by the fact that no clonal relatedness is observed between certain synchronous DCIS-IDC lesions located in different quadrants of the breast.^86,120^ In Pareja et al.’s study, clonal relatedness was not observed between DCIS and IDC foci arising from different mammary quadrants, but was present among lesions in the same quadrant.^86^ In Lip et al.’s recent study, when investigating clonal relationships in 95 paired cases of DCIS and invasive recurrence by advanced single-cell sequencing, they only found 75% (71 out of 95) of the tumor pairs exhibited clear clonal connections while there still remained 18% (17/95) of which displayed no discernible relationship.^97^

Summarily, when earlier studies defined the independent lineage model according to a few discordant markers in DCIS and IDC, which general concordance of other markers might be neglected, was limited by the sample size. Recent extensive genomic analysis of finding a certain proportion of discernible relationships in DCIS and invasive recurrence partially supported the probability of the independent model.

#### Evolutionary bottleneck model

The heterogeneity of breast cancer creates hindrances to targeted therapy and leads to drug resistance. Nevertheless, it facilitates the study of tumor evolution. Previous studies on breast cancer progression have demonstrated a phenomenon of evolutionary bottlenecks in metastases compared with the primary tumor.^124^ Subpopulations of tumor cells from the primary site are enriched in metastases, with or without new mutations that are acquired during the process of metastasis.^125^ Here, the evolutionary bottleneck model for DCIS-IDC transition hypothesizes that during the transition, only a small proportion of DCIS tumor cells with specific genetic events are selected to form a single clone, which subsequently breaks the evolutionary bottleneck and evolves into IDC (Fig. 3b).^113–115^ The evolutionary bottleneck model emphasizes the existence of clonal selection and decreased clonal diversity from DCIS to IDC transition.

Despite the general genetic similarities between synchronous DCIS and IDC, clear differences exist between the two components. In terms of *PIK3CA* mutations in synchronous DCIS and IDC, some studies reported that these mutations were restricted to DCIS,^84,91,100^ while others identified these mutations in IDC but not in DCIS.^100,101^ It may be speculated that the DCIS-IDC transition obeys the: (1) independent lineage model (based on the discordance in PIK3CA mutations between DCIS and IDC), (2) evolutionary bottleneck model, with the selected clone demonstrating clonal shifts of gain or loss *PIK3CA* mutations in IDC, and (3) evolutionary bottleneck model, with the selected clone having *PIK3CA* mutations subsequently developing into the dominant clone in IDC. Besides, based on a small sample size, Doebar et al.’s study selected a subset of 92 invasive tumor-specific variants from 4 synchronous DCIS and IDC lesions, of which 52 variants overlapped between DCIS and IDC lesions, while the other 40 were only restricted to IDC.^126^

In brief, it suggests that clonal selection probably occur during the transition from DCIS to IDC. However, the selected subclones that harbor specific genetic events in DCIS may vary between different patients and new genetic events may even be acquired after DCIS evolving into IDC. In conjunction, these observations support an evolutionary model in which the transition from DCIS to IDC occurs as a result of clonal selection and may obey the rules of Darwinian evolution.^113^

#### Multiclonal invasion model

The multiclonal invasion model differs from the evolutionary bottleneck model in that it refers to multiple subclones escaping and co-migrating to invasive regions to generate IDC, while the evolutionary bottleneck model mainly refers to the dominant subclones selection during the evolution from DCIS to IDC (Fig. 3c).^86,114,115^ Two scenarios are proposed for the multiclonal invasion model. In one scenario, multiple subclones form a relationship of mutual cooperation and even cooperate with the TME. In another scenario, these multiple subclones have different identities and may be considered as “leader” and “follower” subclones, and once the leader subclones break through the basement membrane, their followers join them.^115^ In either scenario, more than one clone may be detected in both DCIS and IDC. Previous studies have mostly examined the model based on genomic evidence, demonstrating highly concordant CNAs and mutations in synchronous DCIS-IDC.^84,91,92,127–130^ However, these studies did not conduct a direct clonal analysis of DCIS and IDC. Recently emerging technologies actualized the tracing of clonal evolution during DCIS progression. In Casasent et al.’s study, a novel technology known as topographic single-cell sequencing was used for analyzing the evolution from DCIS to IDC.^92^ In each matched sample of synchronous DCIS and IDC lesions, they clustered several major clonal tumor subpopulations with highly concordant copy number profiles, which were indicative of stable clonal expansion. Their data showed that in addition to existing in synchronous DCIS and IDC lesions, these subclones shared a common origin in the ducts. By performing deep-exome sequencing in micro-dissected DCIS and IDC, high concordance of nonsynonymous mutations (>87%) were further identified between synchronous DCIS and IDC lesions. These results therefore demonstrated the co-migration of multiple subclones in DCIS to IDC transition, which is in complete contrast to the theory of the bottleneck model. Notably, the shared origin of these subclones from a common ancestor also opposed the independent lineage model.

The multiclonal invasion model may imply that the DCIS-IDC transition: (1) is decided by multiple cancerous cells rather than a specific cell population, and (2) is influenced by noncancerous factors such as TME changes especially in the context of as high genomic concordance between DCIS and IDC.

#### Convergent phenotype model

Another direct lineage model of DCIS progression is the convergent phenotype model.^131^ The model describes that subclones of different genotypes within DCIS may all give rise to an invasive phenotype to establish IDC, with concordant genomic profiles between the DCIS and related IDC (Fig. 3d).^113,114^ This suggests that discordant genotypic tumor cells may undergo potential similar or complementary alterations and finally gain the same invasive phenotype. In particular, the invasive phenotype of IDC may be determined by various combinations of multiple distinct genomic aberrations in DCIS.^113^ This may also explain the negative findings from previous genomic comparisons between DCIS and IDC. Intriguingly, Yates et al. reported two distinct *PTEN* driver mutations appeared in different regions of multifocal DCIS, both of which parallelly evolved into PTEN-null IDC.^132^ Convergent evolution may occur despite genetic divergence acquired during DCIS progression, supporting the presence of mutational diversity in DCIS.

Accumulating evidence suggests that DCIS doesn’t progress with a predetermined pattern in patients, in which both independent and direct lineage models can be observed in different study populations. Thus, the natural pattern of DCIS progression still remains enigmatic. Moreover, microenvironment of the DCIS probably influences the DCIS progression, which may lead to different DCIS progression models. Advanced technologies such as single-cell sequencing and spatial transcriptomics have provided deeper insights into the subclonal dynamics in DCIS progression. During years of follow-up, one or more subclones can persist in the primary lesion and subsequently progress to IDC. These discoveries allow further exploration of prognostic biomarkers for progressive DCIS and corresponding active treatment. The independent DCIS progression model poses new challenges in identifying tumor-intrinsic prognostic biomarkers. In brief, models for DCIS progression remain theoretical, and further research is urgently needed to understand the natural molecular feature of DCIS progression process and the clinical significance relatively.

### Role of the TME in DCIS progression

**Fig. 4:**
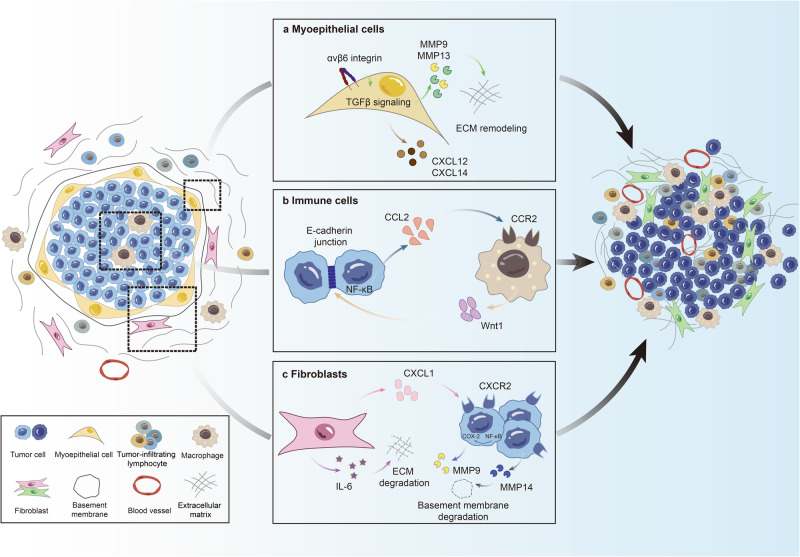
Role of microenvironment in DCIS progression. Tumor-microenvironment crosstalk may facilitate DCIS progression. The environment consists of a range of components including MECs, immune cells, fibroblasts, blood vessels, basement membrane and ECM. **a** MECs: Upregulation of αvβ6 integrin expressed in MECs activates TGFβ signaling, resulting in upregulation of MMP9 and MMP13 and ECM remodeling. Moreover, increased secretion of CXCL12 and CXCL14 from MECs promotes DCIS tumor cell invasion. **b** Immune cells: DCIS cell-derived CCL2 recruit macrophages into the tumor, driving increased Wnt-1 secretion from macrophages, contributing to myoepithelium disruption and the breakdown of E-cadherin junctions. **c** Fibroblasts: Increased secretion of CXCL1 and IL-6 from CAFs drives activation of NF-κB and COX-2 in DCIS cells, which induced and upregulation of MMP9 and MMP14 from DCIS cells, resulting in ECM remodeling and basement membrane degradation. DCIS ductal carcinoma in situ, MECs myoepithelial cells, ECM extracellular matrix, TGFβ transforming growth factor-beta, MMP9 matrix metalloproteinase 9, CXCL12 C-X-C motif chemokine ligand 12, CCL2 C-C motif ligand 2, IL6 interleukin-6, CAFs tumor-associated fibroblasts, NF-κB nuclear factor-kappaB, COX-2 cyclooxygenase 2

Since limited difference have been identified in tumor cells of DCIS and IDC at the genomic or transcriptomic level, many studies have been putting insight into their neighborhood----microenvironment. Emerging evidence suggests that considerable changes in the microenvironment pave the way for DCIS progression.^133^ Notably, different intrinsic subtypes of DCIS have been reported to be associated with distinct tumor microenvironment (TME) and evolutionary pathways to IDC.^21^ In breast cancer, the TME refers to all components surrounding to the cancerous cells in the tumor; it is mainly composed of MECs, immune cells, fibroblasts, extracellular matrix, and blood vessels.^134–136^ Numerous studies have demonstrated that the DCIS-IDC transition is not solely triggered by intrinsic changes in tumor cells; it is also regulated largely by the TME (Fig. 4).^137,138^

#### MECs

In breast ducts, the MECs are located between the epithelium and basement membrane and act as a physical barrier between the epithelium and surrounding stroma.^139^ The MEC layer remains intact in normal ducts, some breast benign lesions, and DCIS. On progression of DCIS, they play a significant role as a physical gatekeeper morphologically, although in some studies they were regarded as an active tumor suppressive factor.^140^

Normal MECs are regarded as natural tumor suppressors due to their anti-tumorigenic, anti-angiogenic, and anti-invasive functions.^141–145^ In this context, they express tumor suppressor proteins including P63, tumor protein p73, laminin 1, and maspin.^80,146,147^ However, DCIS-associated MECs differ from normal MECs. Evidence suggests that DCIS-associated MECs fail to polarize luminal epithelial cells.^148^ Discordant markers have been identified between normal and DCIS-associated MECs on immunohistochemical analysis; these include P63, calponin, CD10, and αvβ6 integrin.^147,149–152^ Notably, Ding et al. found that the functions of normal MECs are partly maintained via an interactive network involving p63 and TCF7 which accomplished by regulation of extracellular matrix proteins and cell adhesion. They further observed a significant decrease of p63 + TCF7+ MECs in MCF10DCIS models compared to normal MECs which correlate with invasive progression.^152^ Thus, the downregulation of tumor suppressor markers and upregulation of pro-invasive markers in DCIS-associated MECs indicate the possibility of achievement of an invasive phenotype, despite the presence of morphological similarities with normal MECs. It is therefore speculated that the progression from DCIS to IDC may be partly attributed to alterations in MECs.

Previous, some in vivo and in vitro studies have demonstrated that the progression from DCIS to IDC is regulated by loss of MEC integrity and function.^140,147,153–155^ Emerging evidence supports the hypothesis that MECs which are conventionally regarded as natural tumor suppressors may act as a promoter during DCIS progression. Upregulation of αvβ6 integrin which expressed in MECs may promote DCIS progression via activation of the transforming growth factor-beta (TGFβ) signaling pathway.^147,156^ The activation of TGFβ signaling has been found to induce the upregulation of matrix metallopeptidase (MMP), specifically MMP9 and MMP13, which play crucial roles in driving the progression of DCIS by contributing to the remodeling of the extracellular matrix and facilitating the invasive properties of DCIS cells.^157,158^ These findings were further supported by clinical observations that elevated MMP13 expression in myoepithelial cells associated with high-grade DCIS cases in Gibson et al.’s study.^158^ In addition, myoepithelial cells in DCIS were found to contribute to tumor-promoting effects through increased expression of C-X-C motif chemokines, such as CXCL12 and CXCL14. Both of which have been shown to promote the migration and invasion of DCIS tumor cells.^159^

#### Immune cells

The immune system is accepted to be a vital component of the TME. A single-cell atlas of breast cancer has revealed the presence of diverse immune cells in the TME including T cells, myeloid cells, macrophages, natural killer cells, and B cells, with T and myeloid cells being the most abundant.^160,161^ Immune cells may suppress tumor growth and metastasis by immune surveillance or drive tumor growth by immunosuppression.^162^ Within the DCIS, the proportion of T cells, B cells, macrophages, and Tregs stands significantly elevated in comparison to the neighboring normal tissue. While within the IDC, the proportion of all T cells (including helper, cytotoxic, and regulatory subtypes), B cells, macrophages, and PD-L1+ immune cells experiences a noteworthy increase in contrast to the adjacent DCIS.^163,164^

Tumor-infiltrating lymphocytes (TILs) have been broadly considered as significant components of the TME. They refer to a cluster of T and B cells that migrate into the tumor and stroma during tumor progression. Previous studies have focused on a subset of TILs mostly having CD3, CD4, CD8, and FOXP3 markers.^165–169^ A higher density of TILs in IDC has been found to be associated with a generally favorable prognosis and better response to adjuvant therapy in clinical trials.^170,171^ In particular, CD8 + T, CD4 + T helper, and CD20 + B cells always indicate good prognosis, while CD4 + FOXP3+ regulatory T cells are proven to drive tumor growth.^172,173^ TILs in DCIS has also been proved to play a potential role in prognostic significance.^164,166^ In comparison to pure DCIS, Toss et al. found that DCIS with synchronous IDC had significantly increased levels of CD8+, CD20+, FOXP3+, PD1+ and PDL1+ cells.^166^ Campbell et al. observed an increased abundance of CD8+, CD4+, CD20+, and FOXP3+ TILs in high-grade DCIS than low-grade DCIS.^174^ Thompson et al. also found increased numbers of CD3+ CD8+, CD4+, CD20+, and FOXP3+ TILs in grade II and III DCIS.^175^ What’s more, DCIS tissue is characterized by a higher abundance of CD4+ helper T cells compared to CD8+ cytotoxic T cells,^175–177^ the opposite is observed in IDC tissue, where cytotoxic T cells outnumber helper T cells.^164^ This discrepancy suggests that IDC exhibits a more pronounced immune-activated environment. There are hypothesis that: (1) greater immune cell activation around the disrupted MEC layer may subsequently trigger further disruption of this layer and result in basement membrane degradation, thereby favoring DCIS progression, or (2) MEC layer disruption and a highly reactive immune system may serve as protective factors against subsequent progression and recurrence.^178^

Macrophages were observed to infiltrate in DCIS. A study by Linde et al. using the MMTV-HER2 model showed that CD206^hi^ macrophages could be drawn to DCIS via NF-κB-driven C-C motif ligand 2 (CCL2) production. The interaction of which subsequently led to heightened Wnt-1 secretion, resulting in myoepithelial disruption and the breakdown of E-cadherin junctions.^179^ More recently, a clinical study found that a high macrophage density in the stroma around DCIS was linked to less favorable outcomes. Specifically, DCIS with a greater density of CD163+ macrophages was indicative of recurrence and ipsilateral invasive recurrence.^180^

In brief, although numerous studies have demonstrated the crucial role of immune cells in DCIS progression, none of the cells could independently predict progression or recurrence alone. The potential mechanisms of DCIS-IDC transition that are regulated by the immune system and cooperate with other factors therefore warrant further evaluation.

#### Fibroblasts

Fibroblasts are the predominant component in the stroma, that produce extracellular matrix and cytokines and respond to the immune system. Fibroblasts also correlate with the polarity and proliferation of epithelium.^172^ Cancer-associated fibroblasts (CAFs) have been proven to play an important role in breast cancer progression. CAFs extracted from IDC differ considerably from those in the normal breast,^181^ and activated fibroblasts (myofibroblasts which express α-smooth muscle actin) have been found in large numbers in IDC. Fibroblasts are proven to promote the DCIS-IDC transition, while MECs suppress progression.^140^

Cytokines, proteases, and growth factors produced by CAFs have been found to facilitate tumor progression, including stromal cell-derived factor 1,^181,182^ TGF-β1,^140,182^ and hepatocyte growth factor.^183,184^ Studies by Hu et al. demonstrated that CAFs promoted DCIS invasion, primarily through triggering NF-κB and COX-2, which leads to an increase in MMP9 and MMP14 expressions,^185^ and further resulted in extracellular matrix (ECM) remodeling and basement membrane degradation respectively.^140^ Utilizing a 3D in vitro model, Osuala et al. delved into the role of interleukin 6 (IL-6) in the DCIS-IDC transition and discovered that CAFs-derived IL-6 initiated DCIS progression partly through cathepsin B-mediated ECM degradation.^186^ Moreover, Sameni et al. demonstrated an interplay between CAFs and myoepithelial cells; CAFs-derived IL-6 enhanced DCIS progression and invasion, while this could be attenuated by myoepithelial cells, partly due to inhibition of CAFs-mediated proteolysis of the extracellular matrix through inhibiting the production of CAFs-derived IL-6.^187^ In Bernard et al.’s study by using MMTV-PyVmT mouse model, they observed that CAFs-derived CXCL1 expression was more pronounced in IDC than in DCIS. CXCL1 produced by CAFs furthered DCIS progression via the CXCR2 receptor and the subsequent activation of various signaling cascades, including the MAPK, NF-κB, Akt, and Stat3 pathways.^188^

Actually, TME is an ecosystem in which progression from DCIS to IDC depends on an evolving spatial distribution and function of multiple cell types, rather than on any single cell subset. The crosstalk between tumor cells and TME components including MECs, immune cells, and fibroblasts synergistically drive DCIS progression. In a recent study, Risom et al. utilized advanced techniques named multiplexed ion beam imaging by time of flight (MIBI-TOF) to construct a spatial cellular map of the progression from DCIS to IDC.^178^ They identified that the myoepithelial layer in DCIS showed less phenotypic diversity and higher proliferation compared to normal tissue. Interestingly, this was accompanied by an increase of stromal CD4 + T cells and mast cells, which subsequently decreased in IDC. Along with the loss of myoepithelium, a greater number of proliferating CAFs and densely aligned fibrillar collagen was found in IDC, however, the potential regulatory mechanisms remain to be discovered. Current advanced techniques may allow investigation of the TME as an integral ecosystem rather than as one of the single-cell subtypes; they may further reveal spatial information and intrinsic interactive mechanisms in the TME.

## Tools for DCIS-IDC research

As the underlying mechanisms of DCIS-IDC transition remain unclear, valuable tools including preclinical models and advanced new technologies are urgently needed for DCIS-IDC research. Both in vitro and in vivo preclinical models have their own advantages and disadvantages, and they should therefore be selected carefully as appropriate. Emerging advanced technologies, such as single-cell sequencing and spatial transcriptomics, have revolutionized our understanding of cancer biology. These powerful tools have provided new insights into DCIS-IDC transition.

### In vitro and in vivo models for research on DCIS progression

**Fig. 5:**
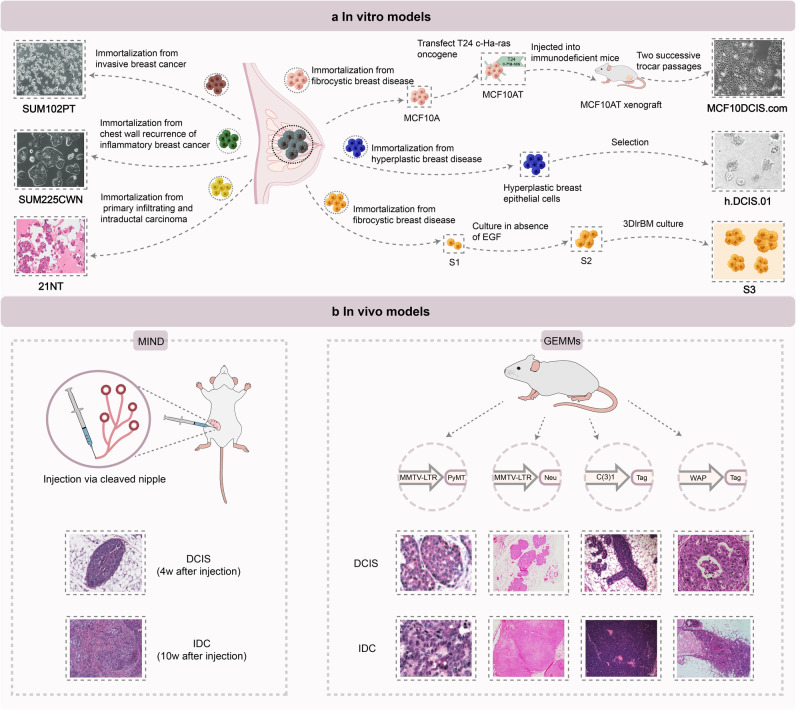
In vitro and in vivo models for DCIS progression research. **a** In vitro models, in which DCIS cell lines that include the MCF10 series,^189^ HMT-3522 series,^191^ 21Tseries,^193^ SUM225CWN,^189^ SUM102PT,^189^ and h.DCIS.01^197^ authentically mimic human DCIS progression for 2D and 3D culture in vitro. **b** In vivo models, in which MIND models^149^ and GEMMs including MMTV-PyMT,^233^ MMTV-Neu,^240^ C3(1)/Tag,^245^ and WAP-T^247^ mouse are available for studying the biology of DCIS in vivo

The study of DCIS-IDC transition has been hindered by lacking of suitable model systems and techniques that recapitulate human DCIS and their progression to IDC. In vitro models mainly include early 2-dimensional (2D) and modified 3D culture models, which allow the study of specific molecular pathways or evaluation of drug efficacy during DCIS progression by controlling experimental conditions. The widely used models including xenografts and genetically engineered mouse models (GEMMs) are suited for studying the biology process of DCIS in vivo. As different models have their own strengths and weakness, it is essential that researchers should choose the appropriate model based on the study aim (Fig. 5).

#### DCIS cell lines

A few DCIS cell lines have been established and utilized in in vitro and in vivo models for the study of DCIS; these include MCF10DCIS.com from the MCF10 series,^189,190^ HMT-3522 series,^191^ 21Tseries,^192,193^ SUM225CWN,^194^ SUM102PT,^195^ and h.DCIS.01 (Fig. 5a).^196,197^

Among these, the MCF10 progression series is regarded as one of the most widely used cell lines in functional studies on DCIS. It was derived from MCF10A, a spontaneously immortalized normal breast epithelial cell line derived from benign fibrocystic breast disease.^198^ A range of cell lines derived from MCF10A provide indispensable model systems that mimic breast cancer progression; these include MCF10AT (proliferation stage),^199^ MCF10DCIS.com (DCIS stage),^190^ and MCF10CA (invasive stage).^200,201^ MCF10DCIS.com is frequently utilized for mimicking human DCIS. Lee et al. used the DCIS.com cell line for constructing in vitro and in vivo models to investigate genes that may regulate DCIS progression. They found that CSTA, FAT1, and DST function as strongly suppressors of DCIS-IDC transition; they also obtained similar results when using the SUM225 and h.DCIS.01 DCIS cell lines.^202^ Maguire et al. observed driver mutations in *TP53* and *PIK3CA* during transformation of the MCF10 progression series (from proliferation stage MCF10AT to invasive stage MCF10CA); this was similar to the findings in primary DCIS and IDC tissues.^203^ These results suggest that the MCF10 series represents a good model for DCIS research. In this context, these DCIS cell lines have been used frequently in both in vivo and in vitro studies on DCIS.

#### 2D culture models

Prior to the use of 3D and in vivo models, conventional 2D cell culture models were the mainstay for breast cancer research.^204^ Studies on DCIS-IDC often use the 2D model to validate specific regulators or pathways that modulate DCIS progression. Compared 3D model, the 2D model offers the advantages of reduced costs, faster results, and easier use.^205^ However, it fails to reflect the natural construction of the tumor which reduces the potential to translate results into 3D, in vivo models, and clinical practice.^206,207^

MCFDCIS cells are widely used for imitating DCIS lesions in 2D models. To explore the role of peroxisome proliferator-activated receptor γ agonists in DCIS progression, Ory et al. used efatutazone, which could activate this pathway in a 2D culture model of the MCFDCIS cell line; the MCFDCIS cells showed an increase in the expression of luminal markers, similar to differentiating MCFDCIS cells. The authors concluded that peroxisome proliferator-activated receptor γ-induced differentiation of MCFDCIS cells could delay the progression from DCIS to IDC.^208^ However, studies have shown that results achieved in 2D models may differ from those obtained in 3D and in vivo models. Li et al. investigated the changes in sensitivity to mitogen-activated protein kinase inhibitors during breast cancer progression; they used the MCF10 cell line series and compared the results between 2D and 3D culture models.^209^ They found the results between these two models to be discordant, with increased sensitivity to these inhibitors in the 3D than in the 2D model. Similarly, Hu et al. identified certain differences between MCFDCIS 2D culture and xenograft models in terms of luminal and myoepithelial markers.^140^ Thus, as the 2D model has certain limitations, studies on DCIS need to employ 3D culture and in vivo models for further investigation.

#### 3D culture models

Unlike the oversimplified 2D models, 3D models attempt to recapitulate the native tumor microenvironment in vitro by building a 3D culture environment that supports cell-cell and cell-matrix interactions.^210,211^ Emerging evidence suggests that the DCIS-IDC transition partially results from interactions between DCIS cells and their TME,^139,140,186^ and that 3D culture models provide a significant in vitro model by co-culturing tumor cells with stromal cells.^187,211–215^ Carter et al. developed a novel 3D culture model that could recapture DCIS using a native physiological bilayer arrangement of myoepithelial and luminal cells; this differed from the traditional use of cell lines to form spheroids.^212^

In summary, 3D models may partly recapitulate the complex TME by co-cultivating multiple cell types and may better mimic the architecture of DCIS in vitro.

#### Mouse-intraductal (MIND) model

The MIND model is a widely used xenograft model,^149,216–220^ which was first developed by Behbod et al.^216^ Two DCIS cell lines or a patient-derived DCIS line were transplanted into the mammary ducts of immunocompromised mice (Fig. 5b). Two DCIS cell lines, namely, MCF10DCIS.com (ER/ progesterone receptor/ HER2-negative) and SUM225 (HER2-positive) formed a basal and HER2-positive subtype xenograft model, respectively; both formed DCIS-like lesions during tumor formation and slowly developed to IDC.^216^ Valdez et al. identified the reproducible growth of patient-derived DCIS in NOD-SCID IL2rγ mice using the MIND model.^217^ In another study, the researchers found that only a proportion of the patient-derived DCIS (54%) line progressed to IDC over a median follow-up of nine months.^218^

Studies using MIND models to investigate the DCIS-IDC transition have reported on the underlying mechanisms of DCIS progression by using candidate promoters or suppressors.^140,149,202,219^ For instance, Elsarraj et al. targeted the B cell lymphoma-9 in a MIND model and found that it acted as a promoter of DCIS.^219^ Compared to other DCIS xenografts (such as xenotransplants of DCIS cell lines or patient-derived DCIS into the mammary fat pad), MIND models better mimic human DCIS lesions with recapitulation of the initial ductal environment.^152,221–223^ However, all these models are limited by the failure to explore the immune effect in DCIS progression (due to their immunodeficient hosts); they do not therefore fully mimic the natural evolution of human DCIS. The artificial construction of DCIS models using human DCIS cells and immunodeficient mice may therefore bias experimental results.

#### Mouse mammary tumor virus-polyoma middle tumor-antigen (MMTV-PyMT) model

Genetically engineered mouse model (GEMMs) are mostly utilized to elucidate the underlying mechanisms of breast cancer biology.^224–227^ Most transgenic models are constructed by targeting tissue-specific oncogenes in mice through tissue-specific promoters.^228,229^ The mammary-specific promoters that are commonly used to produce GEMMs include mouse mammary tumor virus long terminal repeat (MMTV-LTR) and whey acidic protein (WAP) and C(3)1.^229–231^ The MMTV-PyMT model is one of the most widely used GEMMs for breast cancer research. Notably, the MMTV is an important virus that can cause breast cancer in mice. The MMTV-PyMT is a transgenic model, which is generated by overexpression of the PyMT oncogene that is driven by the MMTV-LTR promoter (Fig. 5b).^232^ The MMTV-PyMT model has been demonstrated to undergo four stages during tumor progression (hyperplasia, adenoma/mammary intraepithelial neoplasia, DCIS, and IDC); this is similar to the course of DCIS progression in humans.^233^ The entire course lasts for 10–14 weeks; it generates multifocal tumors and leads to the rapid development of lung metastases. The similarity of pathological and molecular features between MMTV-PyMT and human samples make it a faithful model for research on DCIS progression.^227^

The lack of a natural immune environment in xenograft models makes it impossible to perform experiments pertaining to the immune response for the DCIS initiation and progression, however, the MMTV-PyMT model can compensate for this limitation. Martinez et al. crossed MMTV-PyMT with Foxp3^*DTR*^ knock in mice to establish the regulatory T cell ablation model. They found that regulatory T cell ablation at the DCIS stage resulted in a more aggressive phenotype that promoted DCIS progression.^234^ Boyle and colleagues developed a MMTV-PyMT, C-C chemokine receptor 6-null mouse model and found that the chemokine receptor, CCR6, could promote DCIS initiation and progression by mediating pro-tumorigenic macrophages in the tumor microenvironment.^235^

In summary, the MMTV-PyMT model faithfully reproduces the natural progression of breast cancer with spontaneous tumor initiation. Although the MMTV-PyMT model compensates for weaknesses of the MIND model based on an intact immune system, this model does not authentically mimic human DCIS progression owing to the lack of actual human-derived cells and human tissue microenvironment.^204^

#### MMTV-neu model

MMTV-neu is another GEMM used for modeling breast cancer progression. The prominent ErbB2/HER2/Neu oncogene is associated with breast cancer initiation.^236^ Reports suggest that ErbB2 is overexpressed in nearly 15–20% of breast cancers.^237^ The MMTV-neu model is especially designed for mimicking ErbB2-amplified breast cancer progression.

Notably, the MMTV-neu model was constructed by ErbB2 oncogene overexpression in mice and was driven by the MMTV promoter (Fig. 5b).^238^ Studies have validated that the model can generate pre-invasive disease stage that exhibit histological similarities to human DCIS.^228,236^ Based on this characteristic, Lezzi et al. developed a dynamic mouse model (Balb-NeuT mice) for studying breast cancer progression in vivo. This transgenic model was induced by expressing the r-neuT oncogene driven by MMTV-LTR promoter in BALB/c mice; it underwent transformation from atypical hyperplasia to DCIS, became invasive, and subsequently metastasized.^239^ By using this model, Hosseini et al. found that nearly 80% of metastatic lesions had developed by dissemination from very early lesions (DCIS) rather than those in later stages.^240^ Similarly, Harper et al. found early disseminated cancer cells to be associated with HER2 upregulation on using MMTV-neu mice.^241^

Thus, the MMTV-neu model may be an ideal transgenic model for the study of HER2-positive DCIS. However, it remains unclear whether progression of hyperplasia in the MMTV-neu model can replicate the progression of human HER2-positive DCIS.

#### C3(1)/Tag and WAP-T model

The SV40 large T-antigen (Tag) is an efficient inducer of tumor formation; this is achieved by inactivation of tumor suppressor proteins such as p53 and retinoblastoma protein.^242^ Tag overexpression can lead to formation of breast and prostate cancers that histologically resemble the diseases found in humans.^243^ Similar to the MMTV-PyMT model, the C3(1)/Tag model can mimic human DCIS progression in mice. Green et al. found that the C3(1)/Tag model showed ductal atypia at an age of 8 weeks; it then progressed to DCIS at approximately 12 weeks and to IDC at 16 weeks.^244^ A recent study that used single-cell sequencing showed the existence of pre-DCIS, DCIS, and IDC-like lesions in the C3(1)/Tag model and further demonstrated that DCIS progression was associated with microenvironmental changes.^245^ Comparison of conserved gene expression between the C3(1)/Tag model and human breast cancers revealed that it represented the basal-like subtype found in humans.^245,246^ Another set of transgenic mice, namely, WAP-T mice, were introduced by Schulze-Garg et al. in 2000; they were driven by the SV40 large-T antigen and were induced by the whey acidic protein promoter (Fig. 5b). These mice also develop typical DCIS and IDC-like lesions that morphologically resemble those found in humans.^247^

### New technologies for research on DCIS progression

#### Single-cell sequencing

Single-cell sequencing is a rapidly developing tool.^248^ Bulk sequencing only delineates average biological information of bulk cell populations;^249^ in contrast, single-cell sequencing provides an insight into the single cell level and has revolutionized understanding on tumor biology.

In recent years, single-cell sequencing has made considerable advances in cancer research, with the first single-cell ribonucleic acid (RNA) sequencing (scRNA-seq) study performed in 2009,^250^ the first single-cell deoxyribonucleic acid sequencing conducted in 2011,^251^ and the first single-cell exome sequencing completed in 2012.^252,253^ As intratumor heterogeneity is considerably common in breast cancer,^254–256^ the application of single-cell sequencing is particularly appropriate for breast cancer research.^115,257^ Studies that analyzed clone evolution in breast cancer using single nucleus sequencing,^251^ in which the whole-genome and exome single-cell sequencing^258^ have revealed the clonal relationships among subpopulations.^251^ A recent study by Wang et al. introduced a high-throughput single-cell DNA sequencing technique----Arc-well, specifically designed for the analysis of archival FFPE samples.^98^ While most previous genomic studies of DCIS have been limited to single time-point samples, either utilizing synchronous DCIS-IDC samples or mismatched pairs of DCIS and IDC, Arc-well made the analysis of FFPE sample possible. Arc-well is the first method capable of sequencing FFPE tissue that have been stored for decades, facilitating gene copy number analysis in thousands of single cells. By using this method, the research team conducted a systematic study on primary DCIS and their matched recurrent DCIS or IDC samples. Through this investigation, they unveiled an evolutionary bottleneck model of DCIS progression, providing crucial insights for the treatment of primary DCIS.

Single-cell sequencing has also been used to investigate the role of rare populations in breast cancer progression that could not be detected by prior technologies.^259^ This indicates that it has the potential to identify a rare population of cells in DCIS which may play a pivotal role in promoting DCIS-IDC transition. However, single-cell sequencing has certain limitations; single cells isolated from bulk tissues lose their spatial information, and the process of isolation may influence the cell status (such as causing dissociation-induced gene expression).^260^ In brief, single-cell sequencing has provided a detailed overview of individual cells. However, emerging powerful tools such as multi-omics offer more information than genomic or transcriptomic evaluation^261^ which is expected to bring remarkable benefits for future research on DCIS-IDC transition.

#### Spatial transcriptomics

As scRNA-seq fails to capture in situ spatial information and reflect intercellular communication within tissues, spatial transcriptomics may compensate for these limitations by providing a comprehensive atlas of tissue structure. To date, three main technologies have been developed for detecting spatial transcriptomic information; these include fluorescence in situ hybridization-based, in situ RNA sequencing-based, and spatial barcoding technologies.^262,263^

In addition to scRNA-seq, spatial transcriptomics can be a potent tool for investigating the heterogeneity of cancers.^264–267^ Ståhl and colleagues first introduced the in-situ spatial labeling technology and applied it to breast cancer with synchronous DCIS-IDC components; they found considerable spatial intratumoral heterogeneity among different ductal regions of DCIS. Gene expression was found to be divergent between these regions, probably indicating the different subclones that contributed to DCIS progression.^268^

Spatial transcriptomics can profile spatial information in tissues, but lacks of complete single-cell resolution similar to scRNA-seq. Recent studies have attempted to integrate these two methods to clearly delineate a single cell spatial atlas.^264,266,269,270^ Wei et al. developed a novel computational method, namely, CellTrek to integrate single cell and spatial data;^269^ they further applied CellTrek to two DCIS samples. In one sample, they identified three main tumor subclones; however, the different subclones were mapped to distinct ductal regions, implying extensive spatial intratumor heterogeneity in DCIS. In another sample with synchronous IDC components, they succeeded to map the spatial tumor-immune microenvironment and demonstrated the presence of tertiary lymphoid structures.

In addition to improving understanding on breast cancer biology, spatial transcriptomics can provide new insights into clinical diagnosis of DCIS and IDC and identify predictive markers for DCIS recurrence and treatment.^271,272^ In the study by Yoosuf et al., signatures generated from spatial transcriptomic data of expert-defined DCIS and IDC tissue sections offered highly accurate diagnoses of DCIS and IDC.^272^ In summary, spatial transcriptomics can effectively characterize the gene expression profiles of different cells while retaining the corresponding spatial information, and has considerable potential for providing a detailed spatial map of intercellular communication. However, more advanced technologies are needed for modification.

#### Artificial intelligence

The utilization of artificial intelligence (AI) has presented a viable solution for revolutionizing the interpretation and analysis of histological images. AI possesses the remarkable capability to learn and identify distinctive patterns and interconnections within biological tissues, thereby facilitating a significant transformation in this field.^273^ In DCIS-related studies, utilization of digital image analysis can help pathologists to histologically distinguish DCIS from IDC and differentiate well between different grades of DCIS.^274,275^ Specially designed AI model has been applied to evaluate future breast cancer risk, including the risk of developing DCIS and IDC.^276^ Recently, AI has been rapidly developed in accurately identifying DCIS among other pre-invasive breast lesions as well as in determining the prognostic outcome of DCIS.^273^ Diagnostic disagreements between DCIS and pre-invasive lesions like usual ductal hyperplasia (UDH) and ADH is common in pathologists.^277^ A study performed by Hayward et al. assessed the efficiency of machine learning in differentiating DCIS from atypia, the diagnosis accuracy of which was higher than that of the pathologists while specificity of the feature classification was similar to pathologists.^278^ Moreover, Yamamoto et al. applied a machine learning system to classify four pre-invasive stages of normal tissues, UDH, low and high-grade DCIS solely relying on morphological distinctions in myoepithelial cells, without the need for any information regarding tumor cells.^279^ Given that the distinct composition of the stroma surrounding DCIS compared to normal breast tissue and IDC,^280^ Bejnordi et al. used a deep learning algorithms to distinguish different grades of DCIS and found that the amount of tumor-associated stroma generally increased with higher lesion grade. Interestingly, DCIS with synchronous IDC possessed higher amounts of tumor-associated stroma than pure DCIS.^281^ Klimov et al. conducted an investigation of developing a risk classifier using machine learning-based image analysis on H&E staining of DCIS. Their analysis primarily focused on capturing the spatial relationships between various components of the DCIS, including normal and cancer ducts, lymphocyte region, stroma, and blood vessels. Interestingly, this innovative approach demonstrated remarkable success in accurately predicting the 10-year risk of DCIS recurrence.^282^

Studies to date have exhibited promising advances in applying AI in identifying novel features of DCIS and DCIS progression, which surpassing the dependence of traditional clinicopathological variables. However, it is essential to subject these methods to further testing in extensive patient cohorts with long-term follow-up. Only through rigorous evaluation, the clinical utility of these AI techniques in guiding DCIS management can be ascertained.

## Clinical advancements in DCIS and IDC

### Prognostic biomarkers of DCIS

Approximately 20% of DCIS cases are at risk of future recurrence despite current treatments.^283^ DCIS recurrence is associated with numerous clinical and pathological features.^14,284,285^ In terms of nuclear features, DCIS is classified into three grades, namely, low, intermediate, and high.^14^ Clinically, high-grade DCIS has been demonstrated to possess a significantly higher risk of progression to IDC and subsequent DCIS recurrence than low or intermediate grade DCIS.^7,286,287^ In addition to these well-established risk factors, certain prognostic biomarkers that can predict progression or recurrence and guide treatments are under evaluation; however, they are rarely used in clinical practice.

The ER is a well-established prognostic biomarker and predictor of treatment response in IDC. However, its prognostic and predictive role in DCIS remains unclear. Several studies have reported a significantly lower risk of recurrence in ER-positive DCIS^28,288–292^ compared to ER-negative cases; however numerous studies have found no significant association.^293,294^ Based on the UK/ANZ DCIS trial, a recent study by Thorat et al. used the “clonal method” that scored ER status by clonality of ER expression to analyze the relationship between ER expression and subsequent recurrent events.^290^ The investigators distinguished ER-multi-clonal DCIS (labeled as ER-positive DCIS but showed presence of at least one carcinoma in situ with a complete lack of ER expression) from conventionally classified ER-positive DCIS which lacked complete ER expression in one or more DCIS ducts. Unexpectedly, a similar recurrence risk was found in ER-multi-clonal DCIS and ER-negative DCIS; this was higher than that of ER-completely positive DCIS. This study provided a novel method for determining the ER status more accurately, and avoiding misclassification; this may facilitate evaluation of the actual prognostic value of ER. In this context, a robust association has been found between ER-positvie status and other prognostic factors including lower nuclear grade, smaller tumor size, HER2-negative status, and the absence of comedo necrosis.^293^ Notably, ER expression is higher in low-grade DCIS but lower in high-grade DCIS.^14^

HER2 status is commonly assessed in IDC and is considered an independent prognostic factor for poor outcomes.^295^ However, the predictive value of HER2 for DCIS progression and recurrence remains controversial. Several studies have shown that HER2-positive DCIS is associated with a higher risk of relapse. In clinical trials, HER2-positive DCIS tends to recur in situ, while HER2-negative DCIS has a lower recurrence rate.^19,292,296–298^ Based on these findings, it was supposed that the majority of IDC cases may arise from HER2-negative DCIS (or the HER2-negative subclone of DCIS in cases of heterogeneous HER2 expression). Moreover, HER2 overexpression is more common in pure DCIS than in DCIS with synchronous IDC, suggesting that HER2 expression may not be associated with progression from DCIS to IDC.^20,299,300^ To summarize, HER2-positive DCIS is more likely to relapse, whereas HER2-negative DCIS has a lower recurrence rate. HER2 expression in DCIS is correlated with other poor prognosis indicators such as higher nuclear grade, larger tumor size, ER-negative status, etc.^19,298,301,302^ However, the exact role of HER2 in DCIS initiation and DCIS-IDC transition still remains unclear.

Cyclooxygenase 2 (COX-2) expression is regarded as a potential biomarker in DCIS for the prediction of recurrence; it is also of value as a therapeutic target. COX-2 is highly expressed in both DCIS and IDC, with no significant differences in expression; notably, it is considered to play an important role in early breast cancer carcinogenesis.^303,304^ However, recent studies have demonstrated that COX-2 could be a promising marker for predicting progression from DCIS to IDC.^305^ It was further found that high COX-2 expression in DCIS with large adipocytes predicted a high risk of subsequent IDC.^306^ Other studies have reported that COX-2-positive DCIS may either recur as DCIS or progress to IDC.^307,308^ Notably, COX-2 overexpression in DCIS is also related to the expression of markers indicative of poor prognosis, such as higher nuclear grade, ER-negative status, and HER2-positive status.^14,304^

Ki67 is a predictor of cellular proliferation; high Ki67 expression in DCIS has been demonstrated to be associated with an increased risk of recurrence as either DCIS or IDC.^93,309–311^ An increased risk of subsequent progression to IDC has also been identified in DCIS with combined expression of p16, COX-2, and Ki67.^292,312^

In addition to the above potential protein biomarkers, gene expression detected at the mRNA level also shows prognostic value. The Oncotype DX DCIS score^®^ (Genomic Health) multi-gene panel is the only commercially available tool with a clinically validated signature for prognostication in DCIS. It is a 12-gene panel that includes 7 and 5 cancer-related and reference genes, respectively, and classifies DCIS into low-, intermediate-, and high-risk categories.^313,314^ In addition to predicting the risk of recurrence, it helps to guide treatments. However, the Oncotype DX DCIS score^®^ only predicts the 10-year risk of recurrence after breast-conserving surgery without radiotherapy for primary DCIS; this limits its clinical application to a certain extent.^315,316^ As it has only been validated by the ECOG-ACRIN E5194 trial in a low-risk DCIS patient cohort which met strict criteria, this novel prognostic tool has not been widely adopted.

In summary, the biomarkers discussed previously have not demonstrated good performance as independent predictors of DCIS recurrence and progression. Using a combination of two or more markers may offer better prognostication than an individual marker in certain cases. Most markers correlate with the well-established clinicopathological factors that include age, margin status, tumor size, nuclear grade, and presence of comedo necrosis.^27,28,317^ However, inadequate validation and their uncertain prognostic value have prevented their widespread adoption in clinical practice. The integration of emerging biomarkers and initial clinicopathological factors is expected to offer more reliable stratification of DCIS (based on the risk of recurrence or progression to IDC) in the future.

### Treatment for DCIS

Owing to uncertainties regarding the risk of evolution to IDC or recurrence, current therapeutic approaches for DCIS remain controversial (especially in terms of over- or under-treatment). The primary objective of treatment is to prevent both, potentially progressive DCIS from progressing to malignant IDC and future recurrence. Patients with DCIS commonly undergo surgery and adjuvant therapy. However, there are inaccuracies in DCIS risk stratification and the treatment of DCIS remains controversial. Here, we have summarized all current treatments and related clinical trials for DCIS in Table 2.

**Table 2:** Current treatments and relevant clinical trials for DCIS

	Trial name	Start year	Participant	Median follow-up (year)	DCIS feature	Control arm	Experimental arm	All new breast events (%)	Status
Surgery, radiation therapy	NSABP B-17	1985	818	10.7	/	BCS	BCS + RT	35.1 VS 17.7	Completed
	EORTC 10853	1986	1010	10.5	/	BCS	BCS + RT	27 VS 15	Completed
	SweDCIS	1987	1067	8.0	/	BCS	BCS + RT	32 VS 20	Completed
	UK/ANZ DCIS	1990	1701	12.7	/	BCS (±TAM)	BCS + RT (±TAM)	21.7 VS 9.6	No longer recruiting
	RTOG 9804	1999	636	7.2	Good risk DCIS	BCS (±TAM)	BCS + RT (±TAM)	6.7 VS 0.9	Completed
Endocrine therapy	NSABP B-24	1991	1804	7.0	ER-positive DCIS	BCS + RT+placebo	BCS + RT + TAM	31 VS 20	Completed
	NSABP B-35	2003	3104	9.0	HR-positive DCIS	BCS + RT + TAM	BCS + RT + ANA	7.9 VS 5.8	Completed
	IBIS II DCIS	2005	2980	7.2	HR-positive DCIS	BCS + RT + TAM	BCS + RT + ANA	5 VS 5	Completed
Active surveillance	LORIS	2014	181	/	Low risk DCIS	Surgery	AS	/	No longer recruiting
	COMET	2017	997	/	Low risk DCIS	GCC	AS	/	Active, not recruiting
	LORD	2017	2500^a^	/	Low risk DCIS	GCC	AS	/	Recruiting
	LORETTA	2017	340^a^	/	ER-positive, Low risk DCIS	/	TAM alone	/	Recruiting

*DCIS* ductal carcinoma in situ, *HR* hormone receptor, *ER* estrogen receptor, *BCS* breast-conserving surgery, *RT* radiation therapy, *TAM* tamoxifen, *ANA* anastrozole, *GCC* guideline concordant care, *AS* active surveillance

^a^Estimated participant

#### Surgery and radiation therapy

Mastectomy and breast-conserving surgery are the most two common surgical approaches for DCIS; breast-conserving surgery followed by radiation therapy has been regarded as an acceptable surrogate for mastectomy.^318–320^ Mastectomy may be advised based on risk factors including tumor size, type of DCIS, patient age and preference, and recurrence risk.^321^ For radiation therapy, whole-breast radiation therapy is recommended in a majority of DCIS cases after breast-conserving surgery with the aim of reducing recurrence.^322^

In the clinic, mastectomy is advised for patients with multicentric DCIS or DCIS with microcalcifications scattered along the breast ducts. It remains unclear whether breast-conserving surgery followed by radiation therapy can be advised in these cases.^323–325^ By recruiting all kinds of DCIS, four randomized clinical trials including NSABP B-17, EORTC 10853, SweDCIS, and UK/ANZ DCIS (Table 2) have reported a reduction of local recurrence rate by approximately 50% following breast-conserving surgery plus adjuvant radiotherapy compared to breast-conserving surgery alone^326–330^ The survival benefits of radiation therapy following breast-conserving surgery have been further confirmed. In the RTOG 9804 trial (Table 2), which compared the impact of radiation therapy versus that of observation alone after breast-conserving surgery in good-risk DCIS, the risk of 15-year cumulative overall ipsilateral breast recurrence was found to be 7.1% in the radiation therapy group versus 15.1% in the observation group; the corresponding rates of invasive recurrence were found to be 5.4% versus 9.5%, respectively.^330,331^

#### Endocrine therapy

In ER-positive DCIS, systemic endocrine therapy can reduce both ipsilateral recurrence and contralateral breast cancer, albeit without a significant impact on overall survival.^322,332–334^ In this context, the NSABP B-24 and UK/ANZ DCIS trials (Table 2) evaluated the impact of tamoxifen treatment on recurrence and survival in DCIS.^326,329^ In the NSABP B-24 trial, patients underwent breast-conserving surgery and radiation therapy; this was followed by treatment with either tamoxifen or placebo. The tamoxifen group demonstrated a relatively lower (by 32%) incidence of ipsilateral IDC recurrence compared to the placebo group.^326^ In the UK/ANZ DCIS trial, patients were recruited into a 2 × 2 factorial trial of radiation therapy, tamoxifen, or both treatments after breast-conserving surgery.^329^ The tamoxifen group showed a reduced incidence of both, recurrent ipsilateral DCIS and contralateral new tumors; however, the incidence of recurrent ipsilateral IDC did not differ significantly. Notably, tamoxifen plus radiation therapy failed to show benefit compared with radiation therapy alone. The different results from these two trials may be explained by the younger average age of the participants in the UK/ANZ DCIS trial. Unfortunately, the ER status was not considered in either trial; however, further retrospective analysis of data from the NSABP B-24 and UK/ANZ DCIS trial identified the benefit from tamoxifen to be confined to the ER-positive DCIS group. By contrast, no tamoxifen benefit was found in ER-negative DCIS group.^335,336^

The IBIS-II DCIS and NSABP B-35 trials (Table 2) compared the effect of anastrozole (a non-steroidal aromatase inhibitor) with that of tamoxifen in hormone receptor-positive postmenopausal DCIS patients.^337,338^ They found that anastrozole could be a comparable surrogate for tamoxifen, as it showed a significant improvement in the breast cancer-free interval in postmenopausal population who were younger than 60 years. Both trials found a similar incidence of adverse events with anastrozole or tamoxifen. Despite its effectiveness in reducing recurrent events in hormone receptor-positive DCIS, the side effects of endocrine therapy should be taken into consideration on an individual basis.

#### Other treatments

In order to avoid overtreatment, active surveillance has been tested as an option in low-risk DCIS. Active surveillance refers to the use of regular mammography or other imaging examinations, with intervention only in cases where IDC is detected. Four ongoing prospective randomized trials are comparing active surveillance with standard treatment for low-risk DCIS; these include the LORIS from the UK, LORD from the Netherlands, COMET from the USA, and LORETTA from Japan (Table 2).^339–342^ A computational risk analysis conducted by Ryser et al. compared the disease-specific mortality between active surveillance and usual treatment for DCIS, they concluded that active surveillance could be a viable option for patients with DCIS, especially in older individuals.^343^

The efficacy of anti-HER2-targeted therapy has been established in treating HER2-positive IDC. However, the effectiveness of anti-HER2 therapy in reducing recurrence and improving survival in DCIS remains unclear. A clinical trial previously found that anti-HER2 dendritic cell vaccination could induce a tumor-specific T cell reaction, resulting in improved outcomes in patients with HER2-positive DCIS.^344^ Although HER2 receptor-targeted therapy is the standard in HER2-positive IDC, its role in HER2-positive DCIS needs further validation. The first prospective randomized phase III clinical trial (NSABP B-43) on high-risk HER2-positive DCIS evaluated whether trastuzumab (a HER2 inhibitor) and radiation therapy could reduce recurrence compared to radiation therapy alone (Table 2);^345,346^ the combination failed to meet the target of 36% reduction in ipsilateral breast cancer recurrence and showed a statistically non-significant reduction of 19%. Thus, from current clinical trial results, the use of adjuvant trastuzumab was not supported.

**Fig. 6:**
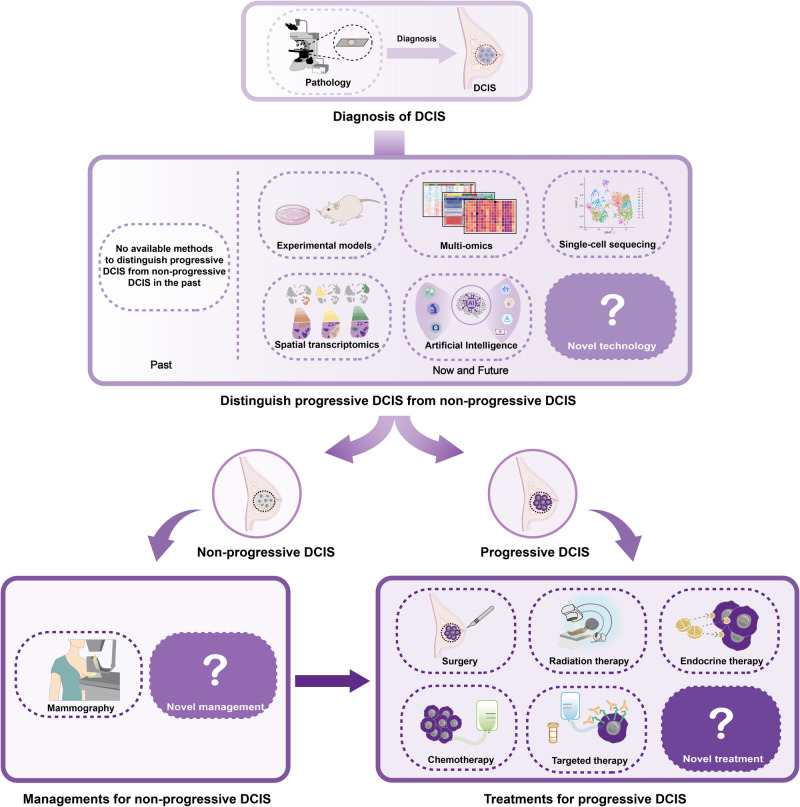
Future management of non-progressive and progressive DCIS. Historically, distinguishing between progressive and non-progressive DCIS in women was challenging, resulting in overtreatment with limited benefits. It is crucial to find a solution to this problem. Advanced technologies, such as experimental models, multi-omics, single-cell sequencing, spatial transcriptomics, artificial intelligence and other emerging technologies, can facilitate the development of a risk stratification system for personalized treatment. Tailoring treatments for non-progressive DCIS differently from progressive DCIS is essential to alleviate the unnecessary burdens associated with overtreatment

In summary, the current treatment and management approaches for DCIS often lead to under- or over-treatment due to a lack of precise risk stratification for patients. Moving forward, there is a need to implement personalized treatment strategies for both non-progressive and progressive DCIS, ensuring that patients receive appropriate and tailored care (Fig. 6).

### Treatment for IDC

Treatment for IDC varies by their molecular subtypes, that include ER-positive, HER2-positive, and TNBC. Typically, a multimodal strategy is employed, combining surgery, radiation therapy, and systemic therapies. Surgical intervention plays a crucial role in the treatment of IDC. Mastectomy and breast-conserving surgery are the two widely used surgical approaches.^347^ Postoperative radiation therapy reduces the recurrence and enhances the survival rates of patients with lymph node involvement and/or who undergo breast-conserving therapy.^348^ Both surgery and radiation therapy are localized treatments for IDC. Nevertheless, systemic therapies, such as endocrine therapy, targeted therapies, chemotherapy, and immunotherapy, have made significant progress in recent years, significantly improving the prognosis of breast cancer. Table 3 presents the representative relevant phase 3 clinical trials of drugs for breast cancers. These drugs have revolutionized the disease trajectory for all subtypes of IDC.

**Table 3:** Phase 3 randomized clinical trials of drugs for breast cancer

Drug	Trial	Participant	Arms	POM	Status	Reference
a. ER-positive breast cancer						
*Aromatase inhibitor*						
Anastrozole	ATAC	9366	Anastrozole vs. tamoxifen vs. anastrozole + tamoxifen	DFS	Completed	ATAC Trialists’ Group.^351^
Anastrozole	TARGET	668	Anastrozole vs. tamoxifen	TTP, OR	/	Bonneterre et al.^385^
Anastrozole	The North American	353	Anastrozole vs. tamoxifen	OR, CR, PR, TTP	/	Nabholtz et al.^386^
Letrozole	/	907	Letrozole vs. tamoxifen	TTP	/	Mouridsen et al.^387^
Exemestane	SOFT and TEXT	5738	Exemestane + OFS vs. tamoxifen + OFS	DFS	Active, not recruiting	Pagani et al.^388^
*CDK4/6 inhibitor*						
Abemaciclib	MONARCH 2	669	Abemaciclib + fulvestrant vs. fulvestrant	PFS	Active, not recruiting	Sledge et al.^389^
Abemaciclib	MONARCH 3	493	Abemaciclib + anastrozole or letrozole vs. anastrozole or letrozole	PFS	Active, not recruiting	Goetz et al.^356^
Palbociclib	PENELOPE-B	1250	Palbociclib (1 yr) + ET vs. ET	iDFS	Completed	Loibl et al.^390^
Palbociclib	PALLAS	5760	Palbociclib (2 yrs) + ET vs. ET	iDFS	Active, not recruiting	Mayer et al.^391^
Palbociclib	monarchE	5637	Abemaciclib + ET vs. ET	iDFS	Active, not recruiting	Johnston et al.^392^
Palbociclib	PALOMA-3	521	Palbociclib+ fulvestrant vs. fulvestrant	PFS	Completed	Cristofanilli et al.^393^
Ribociclib	MONALEESA-2	668	Ribociclib+ letrozole vs. letrozole	PFS	Completed	Hortobagyi et al.^394^
Ribociclib	MONALEESA-3	726	Ribociclib+ fulvestrant vs. fulvestrant	PFS	Completed	Slamon et al.^395^
Ribociclib	MONALEESA-7	672	Ribociclib+ ET vs. ET	PFS	Completed	Lu et al.^396^

b. HER2-positive breast cancer						
*Monoclonal antibody*						
Trastuzumab	HERA	5099	Trastuzumab (2 yrs) vs. trastuzumab (1 yr) vs. observation	DFS	Completed	Piccart-Gebhart et al.^397^
Trastuzumab	NSABP B-31/N9831	3351	Trastuzumab+ chemo. vs. chemo.	DFS	Completed	Romond et al.^398^
Trastuzumab	BCIRG 006	3222	AC → T vs. AC → TH vs. TCH	DFS	Completed	Slamon et al.^399^
Pertuzumab	CLEOPATRA	808	Pertuzumab + trastuzumab + docetaxel vs. trastuzumab + docetaxel	PFS	Completed	Swain et al.^400^
Pertuzumab	APHINITY	4804	Pertuzumab vs. placebo	iDFS	Active, not recruiting	von Minckwitz et al.^362^
Margetuximab	SOPHIA	536	Margetuximab vs. trastuzumab	PFS	Completed	Rugo et al.^401^
*Tyrosine kinase inhibitor*						
Lapatinib	NeoALTTO	455	Lapatinib vs. trastuzumab vs. lapatinib + trastuzumab	pCR	Completed	Baselga et al.^402^
Lapatinib	GeparQuinto	620	Lapatinib+ chemo. vs. trastuzumab+ chemo.	pCR	Completed	Untch et al.^403^
Lapatinib	NSABP B-41	529	Lapatinib+ chemo. vs. trastuzumab+ chemo. vs. lapatinib + trastuzumab+ chemo.	pCR	/	Robidoux et al.^404^
Lapatinib	ALTTO	8381	Trastuzumab vs. lapatinib vs. trastuzumab followed by lapatinib vs. trastuzumab + lapatinib	DFS	Completed	Piccart-Gebhart et al.^405^
Neratinib	NALA	621	Neratinib+ capecitabine vs. lapatinib + capecitabine	PFS	Complete	Saura et al.^406^
Neratinib	ExteNET	2840	Neratinib vs. placebo	iDFS	Complete	Chan et al.^407^
Tucatinib	HER2CLIMB	612	Tucatinib + capecitabine + trastuzumab vs. placebo + capecitabine + trastuzumab	PFS	Complete	Murthy et al.^408^
*Antibody–drug conjugates*						
T-DM1	EMILIA	991	T-DM1 vs. lapatinib + capecitabine	PFS	Complete	Verma et al.^365^
T-DM1	KATHERINE	1486	T-DM1 vs. trastuzumab	iDFS	Active, not recruiting	von Minckwitz et al.^409^
T-DXd	DESTINY-Breast03	524	T-DXd vs. T-DM1	PFS	Active, not recruiting	Cortés et al.^366^

c. TNBC						
*PD1 inhibitor*						
Pembrolizumab	SWOG 1418	1155	Pembrolizumab vs. observation	iDFS	Active, not recruiting	/
Pembrolizumab	KEYNOTE-119	622	pembrolizumab vs. chemo.	OS	Completed	Winer et al.^410^
Pembrolizumab	KEYNOTE-355	847	Pembrolizumab + chemo. vs. chemo.	AEs, PFS, OS	Active, not recruiting	Cortes et al.^411^
Pembrolizumab	KEYNOTE-522	1174	Pembrolizumab + chemo. vs. chemo.	pCR, EFS	Active, not recruiting	Schmid et al.^412^
*PD-L1 inhibitor*						
Atezolizumab	IMpassion031	333	Atezolizumab + chemo. vs. chemo.	pCR	Completed	Mittendorf et al.^413^
Atezolizumab	IMpassion130	902	Atezolizumab + nab-paclitaxel vs. nab-paclitaxel	PFS, OS	Completed	Schmid et al.^383^
Atezolizumab	IMpassion132	572	Atezolizumabe + chemo. vs. chemo.	OS	Active, not recruiting	/
Avelumab	A-Brave	474	Avelumab vs. observation	DFS	Active, not recruiting	/
*PARP inhibitor*						
Olaparib	OlympiA	1836	Olaparib vs. placebo	iDFS	Active, not recruiting	Geyer et al.^414^
Olaparib	OlympiAD	302	Olaparib vs. TPC	PFS	Active, not recruiting	Robson et al.^415^
Veliparib	BROCADE3	509	Veliparib+ carboplatin–paclitaxel vs. carboplatin–paclitaxel	PFS	Active, not recruiting	Diéras et al.^416^
Talazoparib	EMBRACA	431	Talazoparib vs. TPC	PFS	Completed	Litton et al.^417^

*ER* estrogen receptor, *HER2* human epidermal growth factor receptor 2, *TNBC* triple-negative breast cancer, *POM* primary outcome measures, *DFS* disease-free survival, *TTP* time to progression, *OR* objective response, *CR* complete response, *PR* partial response, *OFS* ovarian functional suppression, *PFS* progression free survival, *AEs* adverse event, *iDFS* invasive disease-free survival, *pCR* pathologic complete response, *OS* overall survival, *EFS* event-free survival, *AC* → *T* doxorubicin + cyclophosphamide followed by docetaxel, *AC* → *TH* AC followed by docetaxel + Herceptin, *TCH* docetaxel + carboplatin + Herceptin, *TPC* physician’s choice

#### ER-positive IDC

Endocrine treatment strategies encompass various approaches, including reducing estrogen production, modulating ER signaling, and antagonizing the ER itself.^349^ Among these strategies, the selective ER modulator tamoxifen and aromatase inhibitors (AIs), which inhibit estradiol synthesis, have long been the cornerstone of treatment for ER-positive breast cancer (Table 3).^350^ AIs such as anastrozole, letrozole, fadrozole (available in Japan only), were specifically developed for postmenopausal women. Numerous clinical trials have investigated the efficacy of these drugs in treating postmenopausal patients.^350^ For instance, the ATAC trial evaluated postmenopausal women with early-stage HR-positive breast cancer, randomizing them to receive either anastrozole or tamoxifen. After a median follow-up of 10 years, anastrozole demonstrated superiority over tamoxifen in terms of disease-free survival (DFS), time to recurrence and distant recurrence, although no significant difference was observed in overall survival (OS).^351^ Another meta-analysis conducted by the Early Breast Cancer Trialists’ Collaborative Group (EBCTCG) analyzed the benefits of 5-year treatment with an aromatase inhibitor or tamoxifen in 31,920 postmenopausal women with ER-positive early breast cancer. The analysis revealed that compared to tamoxifen, 5 years of treatment with an aromatase inhibitor reduced the 10-year breast cancer mortality by 15%.^352^ In addition, several randomized trials have aimed to evaluate the efficacy of ovarian function suppression (OFS) and AIs in premenopausal women. The SOFT and TEXT trials compared 5-year treatment with exemestane plus OFS versus tamoxifen plus OFS in premenopausal women with ER-positive early breast cancer. After a median follow-up of 13 years, the combined analysis demonstrated that exemestane plus OFS significantly improved DFS and distant recurrence-free interval, although there was no significant difference in OS compared to the tamoxifen plus OFS regimen.^353^

In the past decade, the significance of utilizing combination therapy in the treatment of ER-positive disease in the metastatic setting has become increasingly evident. Extensive research on the crosstalk between ER signaling and cyclin D1/CDK46 has paved the way for numerous groundbreaking studies. Consequently, the development and approval of CDK4/6 inhibitors have revolutionized the treatment approach for HR-positive metastatic breast cancer (Table 3). Several phase III trials, utilizing CDK4/6 inhibitors such as palbociclib, ribociclib, and abemaciclib in combination with endocrine therapy, have demonstrated remarkable improvements in progression-free survival (PFS) and OS for patients with hormone receptor-positive, HER2-negative metastatic breast cancer.^354–356^

Furthermore, the activation of the PI3K/AKT/mTOR pathway has been identified in ER-positive breast cancer and is often associated with resistance to endocrine therapy. Targeting this pathway has emerged as a potential therapeutic strategy to overcome resistance.^357^ Currently, multiple clinical trials are underway to evaluate the efficacy and safety of various inhibitors, including pan-class I PI3K inhibitors, selective PI3Kα inhibitors, AKT inhibitors, and mTOR inhibitors.^349^

#### HER2-positive IDC

The identification of HER2 as a therapeutic target marked a significant breakthrough in breast cancer treatment. Following the introduction of the first HER2-targeted drug, trastuzumab, in 1990, the survival rates for HER2-positive breast cancer improved dramatically. Previously, the prognosis for HER2-positive breast cancer was similarly gloomy as that for TNBC. Trastuzumab, a humanized monoclonal antibody, binds to the extracellular domain of HER2 and exerts its effects by inhibiting dimerization, suppressing intracellular HER2 signaling through pathways such as MAPK and PI3K/AKT/mTOR, and facilitating antibody-dependent cell-mediated cytotoxicity (ADCC).^358^ Currently, the standard-of-care treatment for HER2-positive breast cancer involves neoadjuvant and adjuvant chemotherapy in combination with anti-HER2 therapy. Pertuzumab, another humanized anti-HER2 monoclonal antibody, with a distinct binding site from trastuzumab, prevents the heterodimerization of HER2 with HER1, HER3, and HER4, ultimately leading to the attenuation of intracellular HER2 signaling.^359,360^ Clinical trials have demonstrated noteworthy outcomes by combining these two monoclonal antibodies with chemotherapy for the treatment of HER2-positive breast cancer. In the CLEOPATRA trial, the combination of pertuzumab, trastuzumab, along with docetaxel, resulted in a median increase of 15.7 months in OS compared to the trastuzumab plus docetaxel only (Table 3).^361^ In another trial, APHINITY, observed similar improvement in invasive DFS in patients who received pertuzumab plus trastuzumab and chemotherapy compared to those who did not receive pertuzumab.^362^

Antibody–drug conjugates (ADCs) drugs have been designed to deliver the cytotoxic effects of chemotherapy specifically to tumor cells (Table 3).^363^ Trastuzumab emtansine (T-DM1) was the first ADC developed for targeting HER2, which comprises trastuzumab linked to the tubulin-binding agent DM1 via a stable thioether linker.^364^ T-DM1 has demonstrated efficacy in women with HER2-positive advanced-stage breast cancer who were previously treated with trastuzumab and a taxane.^365^ Recently, a novel ADC, T-DXd, involving a humanized HER2 antibody with the same sequence as trastuzumab conjugated to deruxtecan (DXd), has shown great activity, including in cases refractory to T-DM1.^366,367^

Other HER2-targeted therapies like tyrosine kinase inhibitors (TKIs), are small molecules that target the intracellular catalytic kinase domain of HER2 (Table 3). Lapatinib, a reversible inhibitor of HER1 and HER2, overcomes trastuzumab resistance in HER2-positive breast cancer.^368^ Neratinib, an irreversible panHER TKI that targets HER1, HER2 and HER4,^369^ improves the 2-year invasive DFS rate compared to placebo when administered after chemotherapy and adjuvant therapy with trastuzumab to women with HER2-positive breast cancer.^370^ Ongoing clinical trials are evaluating pyrotinib, another oral irreversible pan-HER TKI targeting HER1, HER2, and HER4 for the treatment of HER2-positive metastatic breast cancer.^371^ In addition, tucatinib, a HER2-specific TKI capable of crossing the blood-brain barrier, may exhibit activity in patients with brain metastases.^372^

#### TNBC

In TNBC, conventional chemotherapy is standard of care based on an anthracycline and a taxane. However, the toxicity of chemotherapy is a burden on patients and sometimes lack of effectiveness.^373^ More recently, with improvements in understanding of TNBC biology and an increasing appreciation of the potential of personalized therapy strategies, a paradigm shift in the treatment of TNBC is under way. Targeted drugs that intended for BRCA1/2 mutations, intracellular signaling pathways, immune checkpoint have brought great opportunities to improve the prognosis of TNBC patients.^374^

BRCA1/2 gene mutations have been detected in approximately 15–20% of TNBC patients.^31^ These genes play a significant role in the repair of double-stranded DNA through homologous recombination. Tumor cells carrying mutations in BRCA1/2 genes exhibit impaired DNA repair due to deficiencies in homologous recombination repair.^375,376^ Exploiting this vulnerability, PARP inhibitors are utilized to disrupt DNA damage repair, leading to the buildup of excessive DNA damage and consequent elimination of tumor cells in BRCA1/2-deficient TNBC.^377^ Recent clinical trial data have demonstrated that PARP inhibitors (such as olaparib or talazoparib) improved PFS and enhanced quality of life in patients with BRCA-mutated breast cancer when compared to single-agent chemotherapy (Table 3).^378,379^

Immune checkpoint targeting, particularly the programmed death receptor (PD-1) and its ligand PD-L1, has been reported as effective in treating various types of tumors, including melanoma, non-small cell lung cancer, renal cell carcinoma.^380–382^ As TNBC shows heightened genomic instability with abundant immune cell infiltration compared to other breast cancer subtypes, immune checkpoint inhibitors (ICIs) such as pembrolizumab, atezolizumab have demonstrated benefit for TNBC patients (Table 3). Recently, data from clinical trials indicated that the combination of ICIs (especially for PD-1 inhibitor) with chemotherapy in TNBC patients, particularly in those with PD-L1 overexpression, resulted in a higher percentage of pathologic complete response (pCR) and prolonged PFS compared to chemotherapy alone.^383,384^

Ongoing research is exploring additional targeted therapies to inhibit intracellular signaling pathways such as PI3K/AKT/mTOR, EGFR, Notch signaling, and STAT3 signaling, aiming to evaluate their safety and effectiveness in TNBC patients.^374^ However, the current therapeutic options for TNBC are limited compared to ER-positive and HER2-positive breast cancer due to the absence of well-defined targets and the inherent heterogeneity of TNBC itself. As a result, effectively treating TNBC remains a significant challenge that needs to be addressed.

## Conclusion and perspective

The progress and treatment of IDC are advancing rapidly. However, the available treatment options for DCIS remain limited, mainly due to a lack of comprehensive understanding of its underlying mechanisms. While several models, such as the independent lineage model, evolutionary bottleneck model, multiclonal invasion model, and convergent phenotype model, have been proposed in DCIS, there are still more unanswered questions and puzzles that warrant further exploration.

Obviously, in the field of DCIS-IDC research, bottlenecks also persist. Distinguishing between DCIS and early-stage IDC remains a challenge with the present imaging techniques. In addition, predicting which DCIS will transition to IDC versus those that will remain indolent is still a major challenge. As DCIS detection has become more prevalent with advanced screening techniques, there’s a risk of overtreating lesions that might never progress to invasive cancer. While the current treatment recommendations for DCIS are varied, the optimal strategies for management to prevent progression to IDC remain elusive. Furthermore, the biological intricacies governing the shift from DCIS to IDC are yet to be fully unraveled, and the inherent heterogeneity of DCIS further complicate research efforts. Currently, there are already a certain number of tools available for the study of DCIS, such as cells and animal models. However, these tools are still far from sufficient for extensive research. Of course, the use of high-throughput sequencing methods like single-cell sequencing can further uncover the biological characteristics of DCIS.

In the past, we used to focus primarily on the DCIS tumor itself. However, we are now gradually shifting our perspective and recognizing the importance of the surrounding microenvironment in influencing DCIS cells, such as myoepithelial cells, immune cells, fibroblasts. Extensive research has been conducted on the microenvironment of IDC, and drugs targeting the microenvironment to inhibit tumors have emerged. For example, anti-angiogenic drugs like VEGF/VEGFR inhibitors (Bevacizumab, Apatinib) and immune checkpoint inhibitors (Pembrolizumab, Atezolizumab) have demonstrated promising anti-tumor effects.^374^ In the case of DCIS, we are eagerly awaiting research on potential targets for anti-tumor effects specifically focused on the microenvironment.

In clinical practice, there is a great concern for aggressive DCIS because although DCIS is generally considered to have a good prognosis, some patients may experience recurrence or even progression to invasive disease. We aim to identify aggressive DCIS to ensure that they receive adequate treatment, while distinguishing non-aggressive DCIS to spare patients from unnecessary treatments. Prognostic indicators and appropriate treatment strategies are needed, aligning with the concept of precision medicine. As patients’ expectations for survival increase, individualized and precise treatment becomes crucial. A fundamental cornerstone for implementing precision treatment for DCIS patients lies in gaining a clear and in-depth understanding of the underlying biological mechanisms of DCIS and its progression to IDC.

The field of DCIS to IDC research is evolving, with both challenges to overcome and promising avenues to explore. Continued collaboration and innovation in the field will be crucial for improving patient outcomes and understanding the biology of disease progression.
